# Calcium Channel Blocker Versus Renin–Angiotensin System Inhibitor in Risk of Kidney Cancer Among Patients With Hypertension: A Propensity Score‐Matched Cohort Study

**DOI:** 10.1002/cam4.70429

**Published:** 2024-11-16

**Authors:** Minji Jung, Shufeng Li, Zhengyi Deng, Jinhui Li, Mingyi Li, Satvir Basran, Marvin E. Langston, Benjamin I. Chung

**Affiliations:** ^1^ Department of Urology Stanford University Medical Center Stanford California USA; ^2^ Department of Dermatology Stanford University Medical Center Stanford California USA; ^3^ Department of Epidemiology and Population Health, School of Medicine Stanford University Stanford California USA

**Keywords:** antihypertensive drugs, calcium channel blocker, hypertension, kidney cancer, renin–angiotensin system inhibitor

## Abstract

**Background:**

Use of antihypertensive medications could be associated with an increased risk of kidney cancer. Despite their various mechanisms of action, whether this association differs between different classes of medications remains unclear.

**Objective:**

The objective of this study is to compare the risk of kidney cancer between first‐line treatment options of antihypertensive medications in a hypertensive population.

**Method:**

In this retrospective cohort study, we used the MarketScan Databases (2007–2021). We included individuals older than 30 years of age with a diagnosis of hypertension who received first‐line medications for hypertension, which included three classes: angiotensin‐converting enzyme inhibitors (ACEI), angiotensin receptor blockers (ARB), and dihydropyridine calcium channel blockers (dCCB). We applied a propensity score matching method and created three separate cohorts: (1) ARB versus ACEI, (2) dCCB versus ACEI, and (3) dCCB versus ACEI. For non‐dCCB, we repeated the analyses. The primary outcome was kidney cancer incidence. To assess kidney cancer risk, we applied multivariable conditional Cox proportional hazards models.

**Results:**

In the first cohort, ARB use was associated with an increased risk of kidney cancer compared to ACEI use (hazard ratio [HR] 1.10, 95% confidence interval [CI] 1.02–1.18). In the second cohort, dCCB use was associated with an increased risk of kidney cancer compared to ACEI use (HR 1.29, 95% CI 1.18–1.40). In the third cohort, dCCB use was associated with a higher risk of kidney cancer compared to ARB use (HR 1.17, 95% CI 1.08–1.28). Null association was shown when comparing non‐dCCB with ACEI or ARB use.

**Conclusion:**

Use of dCCB showed a higher risk of kidney cancer compared to ACEI or ARB use in patients with hypertension.

## 1 Introduction

Kidney cancer is the sixth most common cancer in men and ninth most common cancer in women [1], and its incidence has been increasing [2, 3]. Due to a lack of nationwide screening and specific signs or symptoms, kidney cancer cases are likely to be detected at a later stage [4], which leads to poorer outcomes with a 5‐year relative survival below 10% [5, 6]. This illustrates the importance of identifying high risk groups and potential preventive strategies for kidney cancer.

Hypertension is an established risk factor for kidney cancer development [3, 4, 7]. In the United States, it is estimated that ~50% of adults (119.9 million) have hypertension in 2023. The hypertensive population is on the rise, driven by an aging population, along with the rising prevalence of obesity [8]. Of those, 80% (94.9 million) are recommended to take antihypertensive medications to control their blood pressure and prevent cardiovascular diseases [8]. Due to the chronic nature of hypertension, it is common for individuals to take antihypertensive medications throughout their entire lives.

Although antihypertensive medications are necessary to control blood pressure, evidence from preclinical experimental studies supporting a higher risk of kidney cancer with antihypertensive medication use has been growing. They have demonstrated that various classes of antihypertensive medications could potentially contribute to developing kidney cancer through different carcinogenic mechanisms, such as facilitating tumor cell proliferation, angiogenesis, or migration [9, 10, 11]. Considering their distinct mechanisms of actions in controlling blood pressure [12], it is reasonable to hypothesize that each class of these medications may have varying effects on kidney cancer development. To accurately evaluate these differential influences, it is necessary to conduct a direct comparison between different classes. To date, however, research in this area has been limited to case–control studies, which compared patients who took mediations to those who did not take them [13, 14, 15, 16]. It is also important to emphasize that the medications are prescribed not only for hypertension but also for other conditions such as heart failure and arrhythmia, which was not considered in previous studies. These significant limitations are more likely to introduce several biases (e.g., indication bias, selection bias, prevalent user bias).

Given the widespread use of antihypertensive medications and their potentially distinct roles in cancer, we aimed to directly compare the risk of kidney cancer among first‐line antihypertensive medication options–angiotensin‐converting enzyme inhibitor (ACEI), angiotensin receptor blocker (ARB), dihydropyridine calcium channel blocker (dCCB), and non‐dCCB–in a hypertensive population. We conducted five head‐to‐head comparison analyses using propensity score matching: (1) ACEI versus ARB, (2) dCCB versus ACEI, (3) dCCB versus ARB, (4) non‐dCCB versus ACEI, and (5) non‐dCCB versus ARB. This approach addresses previous research limitations by considering the distinct mechanisms of these drugs and minimizing potential biases, leading to more accurate results.

## 2 Materials and Methods

### 2.1 Data Source and Study Design

This retrospective cohort study was conducted using the Merative MarketScan Commercial and Medicare Databases (2007–2021, version 3.0) [17]. The Databases include more than 190 million commercially insured individuals with employer‐sponsored health insurance or individuals over the age of 65. These include de‐identified individuals' longitudinal health insurance claim information including socio‐demographics, insurance enrollment, medical institution visits, diagnostic codes for inpatient and outpatient settings, and records of prescription claims for outpatient setting. The diagnoses were recorded according to the International Classification of Diseases (ICD), 9th and 10th revision. The prescription claims were recorded according to generic drug names with the National Drug Codes. This study using the de‐identified data, hosted by the Stanford Center for Population Health Sciences (PHS), is covered under the PHS umbrella IRB 40974.

### 2.2 Study Population

We included patients with a diagnostic record of hypertension who initiated first‐line treatment options of antihypertensive medications [12]. Diagnosis of hypertension was identified using ICD‐9 and ICD‐10 codes (Table S1). To reduce potential indication bias, the first‐line treatment options for hypertension, which are the most commonly used three classes, included: renin–angiotensin–aldosterone system inhibitors including ACEI and ARB, and dCCB [12]. We repeated the main analyses for non‐dCCB. Due to the diverse indications of beta‐blockers (BB) and diuretics (DU), we considered them as covariates in this study. We defined the index date as the first prescription date of the medications. We excluded the following patients: (1) those under the age of 30 at the index date [18]; (2) those with a history of any cancers except for non‐melanoma skin cancer prior to the index date; (3) those with a history of Von Hippel–Lindau, tuberous sclerosis, or polycystic kidney disease prior to the index date; (4) those with missing data of age and sex at the index date; and (5) those without insurance enrollment within 1 year before and after the index date.

To perform the head‐to‐head comparison analyses, we assembled three main separate study cohorts: (1) ARB versus ACEI, (2) dCCB versus ACEI, and (3) dCCB versus ARB. When comparing ACEI and ARB, we selected ACEI as a comparator because ACEI has been used more commonly than ARB [12]. When comparing dCCB with ACEI or ARB, we selected ACEI or ARB as an active comparator because ACEI and ARB have similar pathophysiological mechanisms and clinical effects by inhibiting renin–angiotensin–aldosterone system [12].

### 2.3 Index Antihypertensive Medications

The index antihypertensive medication, the main exposure, was defined as an initiation of ACEI, ARB, or dCCB (Table S2) [12]. To ascertain their initiation, we included individuals with no previous prescription record of the index medication prior to the index date. To ensure their long‐term use, we included individuals who continued longer than 180 consecutive days with at least 80% adherence using the proportion of days covered (PDC) [19]. The PDC was calculated by dividing the total number of days the medication was supplied by the total prescribed duration, from the first to the end date of the last prescription.

To assess whether the associations were different according to medication exposure, we identified a cumulative dose of defined daily dose (cDDD) and duration of antihypertensive medication use. The DDD is the assumed average maintenance dose per day for a medication for its main indication in adults [20]. The cDDD was calculated as a ratio of “total exposure amount” to the DDD value of the medication. The “total exposure amount” was calculated by multiplying the cumulative dose of the medication by the number of days' supply covered by the medication. As this provides a fixed unit of measure independent of strength or medication type, we were able to compare medication exposure between different classes. We categorized the cDDD as four groups: < 500, 500–1500, 1500–3000, and more than 3000 cDDD. We classified the duration as six groups: < 1, 1–3, 3–5, 5–7, 7–10, and longer than 10 years.

### 2.4 Other Antihypertensive Medications

In addition to the index medications, we identified the other antihypertensive medications, which included three classes: BB, DU, and non‐dCCB [12]. BB and DU are more likely to be added to the index medication or used for other compelling indications besides hypertension, such as atrial fibrillation or heart failure [12]. We identified the use of these medications during 1 year prior to the index date. For non‐dCCB, we repeated the analyses to assess the risk of kidney cancer. Non‐dCCB shares a similar mechanism of action with dCCB, by antagonizing calcium channels, but has different primary target tissues: dCCB primarily targets blood vessels, whereas non‐dCCB affects heart. We assembled two additional study cohorts: (4) non‐dCCB versus ACEI and (5) non‐dCCB versus ARB. Similar to the main analysis, ACEI and ARB was used as an active comparator, respectively.

### 2.5 Study Outcomes

The study outcome was kidney cancer incidence, identified with at least one hospitalization or discharge diagnosis code or two outpatient diagnosis codes (ICD‐9 codes, 189.0 and ICD‐10 codes, C64). To ensure an incident case, we excluded prevalent cases before the index date. Each hypertensive patient was followed from the index date to the study outcome occurrence, disqualification of the insurance enrollment, or the end date of the study period (December 31, 2021), whichever came first. Given the chronic use of antihypertensive medications, we used an intention‐to‐treat approach in the main analysis, implying that treatment termination was disregarded. We assumed that the index medication may affect the risk of cancer, which is a long‐term progressive disease with a long latency period, even after termination of treatment. In a sensitivity analysis, we used an as‐treated approach. This approach accounted for a termination of treatment such as discontinuation or switch, which was defined as no claim filed for the medication within 6 or more months after the due date of a refill prescription. In this case, individuals were followed up by the outcome occurrence, termination of treatment, disqualification of the insurance enrollment, or the end date of the study period, whichever came first.

### 2.6 Potential Confounders

We considered the following variables as potential confounders: (1) demographic variables including age, sex, geographical region of individuals' residence, and insurance type at the index date; (2) hypertensive complications or comorbidities including myocardial infarction, ischemic stroke/transient ischemic stroke, hemorrhagic stroke, diabetes mellitus (DM) with or without complications, heart failure, angina, atrial fibrillation, dyslipidemia, peripheral vascular disease, chronic kidney disease (CKD), renal failure, and chronic obstructive pulmonary disease (COPD) during 1 year prior to the index date (Table S1); and (3) other medication use including lipid‐lowering agents, anti‐diabetic agents, anti‐thrombotic agents, anti‐platelet agents, and anti‐analgesic agents during 1 year before the index date [12, 21]. Charlson comorbidity index (CCI) was calculated to estimate the burden of individual's health condition [22].

### 2.7 Statistical Analysis

We used propensity score (PS) matching in a 1:1 ratio using caliper without replacement to minimize the imbalance of potential confounders. Multivariable logistic regression models were used to estimate PS conditionally on the abovementioned variables including demographics, comorbidities, and medications. The caliper was calculated as 0.1 of the standard deviation of the logit of PS. To assess the statistically significant differences between PS‐matched groups, we used absolute standardized mean differences with a cutoff value of > 0.1 [23]. We calculated cumulative incidence during the follow up and incidence rates per 100,000 person‐years. We examined the proportional hazards assumptions using the Schoenfeld residual method and conducted the conditional Cox proportional hazards regression. To reduce potential confounding effects, we further adjusted the model for CCI, which was not used in the matching model.

To assess whether the associations differ according to the baseline characteristics, we conducted several subgroup analyses according to age, sex, comorbidities, and the previous use of other antihypertensive medications. To confirm the robustness of the main findings, we conducted six sensitivity analyses. First, to examine the impact of possibly residual indication bias, we redefined the use of index antihypertensive medications as an initiation with no prescribed record of any other antihypertensive medications, such as BB or DU, prior to the index date. Second, to assess the effects of long‐term use, we restricted the study patients who took antihypertensive medications more than 1000 cDDD [13]. Third, to allow for an induction period of kidney cancer related to antihypertensive medication exposure and to minimize reverse causation that hypertension caused by undiagnosed kidney cancer, we applied 2 years of lag time after the index date. Fourth, to reduce the potential remaining indication bias, we repeated the analyses by excluding patients who had a history of potential compelling indications such as myocardial infarction, ischemic stroke, hemorrhagic stroke, DM, heart failure, angina, atrial fibrillation, peripheral vascular disease, CKD, or renal failure [12]. Fifth, to account for discontinuation of the index medication or switch to another medication, we applied the as‐treated approach as mentioned above. In such case, we assumed that the medication may not affect the cancer risk after termination of treatment. Lastly, we repeated the analyses including the entire population before PS matching to examine the associations independently with unknown probabilities of receiving different antihypertensive medications. For sensitivity analysis, to account for potential imbalance between two groups, the Cox regression model was adjusted for all confounders, which were used in the matching model in the main study. We set up statistical significance as a two‐tailed *p*‐value < 0.05. All data analyses were conducted using SAS Version 9.4 and R Version 4.0.

## 3 Results

### 3.1 Baseline Characteristics

After applying our inclusion and exclusion criteria, the first cohort included patients who received ACEI (*n* = 640,671) and ARB (*n* = 640,671); the second cohort included those with ACEI (*n* = 373,601) and dCCB (*n* = 373,601); and the third cohort included those with ARB (*n* = 408,491) and dCCB (*n* = 408,491) (Figure S1). Overall, baseline characteristics were well‐balanced between the groups (Table 1). Across the three cohorts, ~50% were over 60 years old, women, and lived in the Midwest. In this hypertensive population, dyslipidemia accounted for the highest proportion of comorbidities (< 60%), followed by DM with or without complications (< 30%).

**TABLE 1:** Baseline characteristics of the three study cohorts.

	Study cohort 1				Study cohort 2				Study cohort 3			
	ACEI		ARB		ACEI		dCCB		ARB		dCCB	
	*N* = 640,671		*N* = 640,671		*N* = 373,601		*N* = 373,601		*N* = 408,491		*N* = 408,491	
	*N*	%	*N*	%	*N*	%	*N*	%	*N*	%	*N*	%
Age (median, IQR)	58 (50–64)		58 (51–64)		59 (51–65)		58 (51–65)		59 (51–65)		59 (51–65)	
< 50 years	144,101	22.5	135,786	21.2	81,955	21.9	83,009	22.2	85,963	21.0	90,333	22.1
50–60 years	217,283	33.9	216,302	33.8	119,266	31.9	118,960	31.8	131,206	32.1	128,997	31.6
60–70 years	180,868	28.2	186,666	29.1	105,326	28.2	104,625	28.0	117,934	28.9	114,677	28.1
≥ 70 years	98,419	15.4	101,917	15.9	67,054	17.9	67,007	17.9	73,388	18.0	74,484	18.2
Sex												
Men	323,866	50.6	319,898	49.9	190,213	50.9	192,239	51.5	206,499	50.6	210,215	51.5
Women	316,805	49.4	320,773	50.1	183,388	49.1	181,362	48.5	201,992	49.4	198,276	48.5
Geographical region												
North	90,221	14.1	109,974	17.2	56,319	15.1	62,482	16.7	71,182	17.4	66,663	16.3
Northeast	168,934	26.4	134,071	20.9	99,899	26.7	89,746	24.0	90,173	22.1	100,375	24.6
Midwest	278,353	43.4	291,746	45.5	156,456	41.9	164,263	44.0	176,602	43.2	179,594	44.0
South	91,451	14.3	94,246	14.7	55,686	14.9	52,981	14.2	65,632	16.1	57,010	14.0
West	11,712	1.8	10,634	1.7	5241	1.4	4129	1.1	4902	1.2	4849	1.2
Insurance type												
HMO	79,141	12.4	74,211	11.6	45,566	12.2	50,346	13.5	49,726	12.2	54,327	13.3
PPO	356,146	55.6	354,171	55.3	203,949	54.6	196,816	52.7	220,386	54.0	217,070	53.1
Others	205,384	32.1	212,289	33.1	124,086	33.2	126,439	33.8	138,379	33.9	137,094	33.6
Duration of hypertension diagnosis to medication initiation, days (median, IQR)^a^	548 (367–1313)		570 (325–1532)		630 (430–1559)		655 (480–1862)		620 (420–1875)		643 (510–1785)	
Previous use of other AHTN												
BB	84,651	13.2	81,243	12.7	55,188	14.8	52,330	14.0	55,484	13.6	58,084	14.2
DU	103,786	16.2	99,682	15.6	63,087	16.9	59,157	15.8	72,544	17.8	67,544	16.5
Thiazide DU	63,587	9.9	60,799	9.5	38,245	10.2	39,524	10.6	44,457	10.9	45,207	11.1
Loop DU	25,619	4.0	25,298	3.9	14,936	4.0	11,301	3.0	17,830	4.4	13,837	3.4
K+ sparing DU	22,354	3.5	21,192	3.3	14,043	3.8	12,336	3.3	15,207	3.7	13,304	3.3
Comorbidities												
Dyslipidemia	374,562	58.5	378,294	59.0	210,265	56.3	209,866	56.2	238,649	58.4	233,825	57.2
DM w complications	44,610	7.0	47,801	7.5	27,225	7.3	27,849	7.5	32,533	8.0	33,111	8.1
DM w/o complications	157,135	24.5	162,352	25.3	82,386	22.1	83,077	22.2	93,724	22.9	95,586	23.4
PVD	42,167	6.6	43,622	6.8	28,174	7.5	28,208	7.6	32,243	7.9	32,370	7.9
Ischemic stroke/TIA	41,142	6.4	41,508	6.5	28,973	7.8	28,542	7.6	30,117	7.4	31,549	7.7
Hemorrhagic stroke	2859	0.4	2717	0.4	2604	0.7	2739	0.7	2066	0.5	2532	0.6
Myocardial infarction	18,872	2.9	18,853	2.9	11,424	3.1	11,509	3.1	13,149	3.2	13,022	3.2
Angina	6156	1.0	6181	1.0	4910	1.3	5208	1.4	5883	1.4	5995	1.5
Heart failure	31,109	4.9	31,057	4.8	17,455	4.7	16,736	4.5	19,832	4.9	19,565	4.8
Atrial fibrillation	31,836	5.0	32,168	5.0	16,934	4.5	15,849	4.2	18,611	4.6	18,059	4.4
Chronic kidney disease	28,703	4.5	31,181	4.9	20,996	5.6	23,024	6.2	27,901	6.8	29,109	7.1
Renal failure	6241	1.0	6641	1.0	4561	1.2	5176	1.4	5720	1.4	6335	1.6
COPD	38,696	6.0	39,074	6.1	24,236	6.5	23,197	6.2	26,485	6.5	26,413	6.5
CCI (median, IQR)	2 (1–3)		2 (1–3)		2 (1–3)		2 (1–3)		2 (1–3)		2 (1–3)	
Other medication use												
Lipid‐lowering agents	116,555	18.2	113,138	17.7	61,430	16.4	61,422	16.4	71,325	17.5	71,171	17.4
Anti‐diabetic agents	51,261	8.0	50,321	7.9	19,700	5.3	20,412	5.5	24,711	6.0	25,481	6.2
Anti‐thrombotic agents	14,810	2.3	14,707	2.3	8029	2.1	7635	2.0	8741	2.1	8740	2.1
Anti‐platelet agents	19,594	3.1	19,376	3.0	12,262	3.3	12,424	3.3	13,773	3.4	14,063	3.4
Anti‐analgesic agents	56,999	8.9	56,301	8.8	34,207	9.2	33,133	8.9	35,831	8.8	35,750	8.8

Abbreviations: ACEI, angiotensin‐converting enzyme inhibitor; AHTN, antihypertensive drugs; ARB, angiotensin receptor blocker; BB, beta‐blocker; CCI, Charlson comorbidity index; COPD, chronic obstructive pulmonary disease; dCCB, dihydropyridine calcium channel blocker; DM, diabetes mellitus; DU, diuretic; HMO, Health Maintenance Organization; IQR, interquartile range; PPO, preferred provider organization; PVD, peripheral vascular disease; TIA, transient ischemic attack.

^a^ Duration of hypertension was calculated as time period (days) from the first diagnosis of hypertension to the first prescription date of index medication.

### 3.2 Main Analyses

Table 2 summarizes the results of the main analysis. In the first cohort, when comparing ARB with ACEI use, 1551 (0.24%) and 1578 (0.25%) kidney cancer events occurred in ACEI and ARB groups, respectively. ARB use was associated with an increased risk of kidney cancer compared with ACEI use (adjusted hazard ratio [HR] 1.10, 95% confidence interval [CI] 1.02–1.18). In the second cohort, when comparing dCCB with ACEI use, 894 (0.24%) and 1091 (0.29%) kidney cancer events occurred in ACEI and dCCB groups, respectively. The use of dCCB showed a higher risk of kidney cancer compared with ACEI use (HR 1.29, 95% CI 1.18–1.40). In the third cohort, when comparing dCCB with ARB use, 964 (0.24%) and 1166 (0.29%) kidney cancer events occurred in ARB and dCCB groups, respectively. The use of dCCB had an elevated risk of kidney cancer compared with ARB use (HR 1.17, 95% CI 1.08–1.28).

**TABLE 2:** Risks of kidney cancer associated with the use of ACEI, ARB, and dCCB in the three cohorts.

Study cohort 1	ACEI (*N* = 640,671)				ARB (*N* = 640,671)				ARB vs. ACEI	
	Event, *N*	%	PY	IR per 100,000 PY	Event, *N*	%	PY	IR per 100,000 PY	Unadjusted HR (95% CI)	Adjusted HR^a^ (95% CI)
	1551	0.24	2,062,283.4	75.2	1,578	0.25	2,070,866.1	76.2	1.10 (1.03–1.18)	1.10 (1.02–1.18)

Study cohort 2	ACEI (*N* = 373,601)				dCCB (*N* = 373,601)				dCCB vs. ACEI	
	Event, *N*	%	PY	IR per 100,000 PY	Event, *N*	%	PY	IR per 100,000 PY	Unadjusted HR (95% CI)	Adjusted HR^a^ (95% CI)
	894	0.24	879,996.0	101.6	1,091	0.29	913,761.4	119.4	1.30 (1.19–1.42)	1.29 (1.18–1.40)

Study cohort 3	ARB (*N* = 408,491)				dCCB (*N* = 408,491)				dCCB vs. ARB	
	Event, *N*	%	PY	IR per 100,000 PY	Event, *N*	%	PY	IR per 100,000 PY	Unadjusted HR (95% CI)	Adjusted HR^a^ (95% CI)
	964	0.24	957,562.9	100.7	1,166	0.29	964,669.1	120.9	1.18 (1.08–1.28)	1.17 (1.08–1.28)

Abbreviations: ACEI, angiotensin‐converting enzyme inhibitor; ARB, angiotensin receptor blocker; CI, confidence interval; dCCB, dihydropyridine calcium channel blocker; HR, hazard ratio; IR, incidence rate; PY, person‐years.

^a^ The model was adjusted for Charlson comorbidity index.

### 3.3 Subgroup and Sensitivity Analyses

**FIGURE 1:**
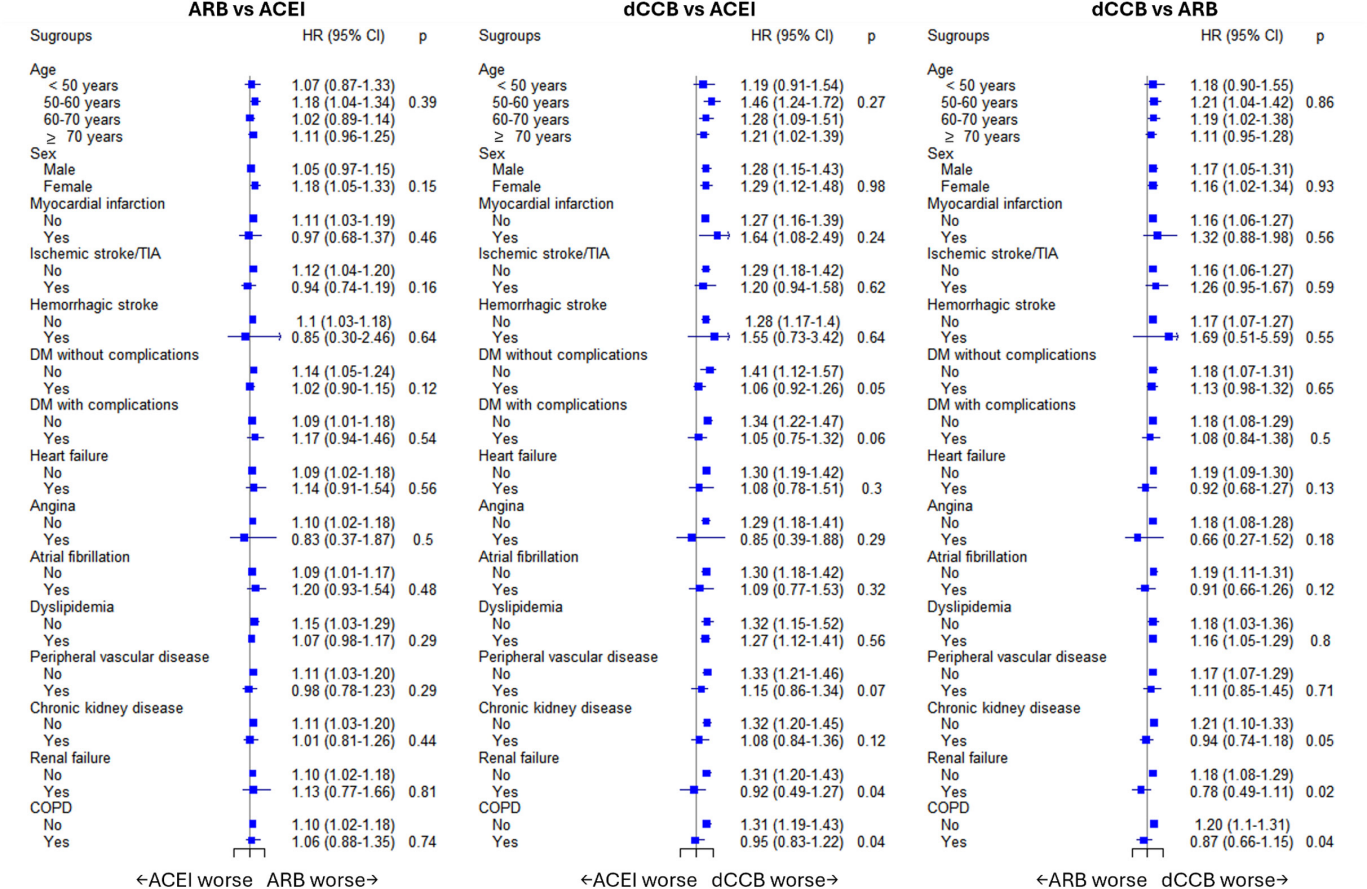
Forest plots of subgroup analyses for kidney cancer risk associated with ACEI, ARB, and dCCB. Across the three cohorts, results from the subgroup analyses generally supported the main findings. In the second and third cohorts, a greater risk was observed among patients without renal failure/COPD than those with renal failure/COPD. The model was adjusted for Charlson comorbidity index. ACEI, angiotensin‐converting enzyme inhibitor; ARB, angiotensin receptor blocker; CI, confidence interval; COPD, chronic obstructive pulmonary disease; dCCB, dihydropyridine calcium channel blocker; DM, diabetes mellitus; HR, hazard ratio; PVD, peripheral vascular disease; TIA, transient ischemic attack.

**FIGURE 2:**
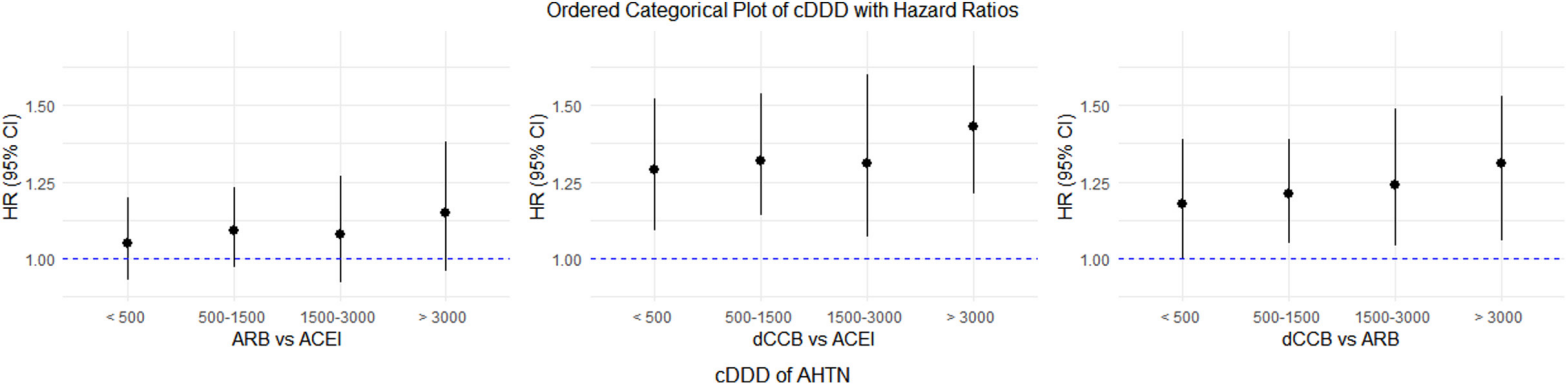
Plots of exposure to antihypertensive medications with the risk of kidney cancer. According to the medication exposure, consistent trends supporting the main finding were shown. Stronger hazard ratios were observed in the highest exposure group across the three study cohorts. The hazard ratios were adjusted for Charlson comorbidity index. ACEI, angiotensin‐converting enzyme inhibitor; ARB, angiotensin receptor blocker; cDDD, cumulative defined daily dose; CI, confidence interval; dCCB, dihydropyridine calcium channel blocker; HR, hazard ratio.

Across the three cohorts, results from the subgroup analyses supported the main findings (Figure 1 and Tables S3–S6). When it comes to renal failure, when comparing dCCB to ACEI use, a greater risk was observed among patients without renal failure than those with renal failure (*p* for difference = 0.04). When comparing dCCB to ARB use, similar results were observed among patients without and with renal failure (*p* for difference = 0.02). Similarly, compared to ACEI or ARB use, the use of dCCB showed a higher risk of kidney cancer among patients without COPD. Among those without COPD, null associations were observed when compared dCCB use to ACEI or ARB use. According to the medication exposure, consistent results supporting the main findings were shown with a trend toward a higher cDDD associated with an increased risk of kidney cancer (Figure 2 and Table S7). The results from the sensitivity analyses showed robust results, regardless of constructing different study populations, applying a lag time, or considering termination of treatment (Table 3).

**TABLE 3:** Sensitivity analyses for kidney cancer risk associated with the use of ACEI, ARB, and dCCB.

Study cohort 1	ACEI			ARB			ARB vs. ACEI
	*N*	Event, *N*	%	*N*	Event, *N*	%	Adjusted HR (95% CI)^a^
Main analysis	640,671	1551	0.24	640,671	1578	0.25	1.10 (1.02–1.18)
Redefinition of new AHTN use	479,635	1095	0.23	484,205	1161	0.24	1.11 (1.02–1.20)
Long‐term use (1000+ cDDD)	185,898	663	0.36	186,808	707	0.38	1.12 (1.02–1.25)
Lag time (2 years)	640,671	870	0.14	640,671	853	0.13	1.11 (1.01–1.22)
Without compelling indications	402,487	742	0.18	395,218	796	0.20	1.14 (1.03–1.26)
As‐treated approach	640,671	1019	0.16	640,671	1137	0.18	1.03 (0.96–1.11)
Without propensity score matching	816,301	1882	0.23	726,045	1896	0.26	1.11 (1.04–1.20)

Study cohort 2	ACEI			dCCB			dCCB vs. ACEI
	*N*	Event, *N*	%	*N*	Event, *N*	%	Adjusted HR (95% CI)^a^
Main analysis	373,601	894	0.24	373,601	1091	0.29	1.29 (1.18–1.40)
Redefinition of new AHTN use	255,662	535	0.21	256,908	729	0.28	1.35 (1.21–1.52)
Long‐term use (1000+ cDDD)	98,532	360	0.37	101,830	480	0.47	1.30 (1.15–1.47)
Lag time (2 years)	373,205	498	0.13	373,017	507	0.14	1.20 (1.06–1.28)
Without compelling indications	235,364	415	0.18	235,753	560	0.24	1.38 (1.22–1.64)
As‐treated approach	373,601	563	0.15	373,601	850	0.23	1.16 (1.07–1.26)
Without propensity score matching	749,990	1616	0.22	470,348	1385	0.29	1.30 (1.20–1.41)

Study cohort 3	ACEI			dCCB			dCCB vs. ARB
	*N*	Event, *N*	%	*N*	Event, *N*	%	Adjusted HR (95% CI)^a^
Main analysis	408,491	964	0.24	408,491	1166	0.29	1.17 (1.08–1.28)
Redefinition of new AHTN use	242,542	566	0.23	245,700	683	0.28	1.17 (1.05–1.32)
Long‐term use (1000+ cDDD)	107,484	406	0.38	105,934	511	0.48	1.22 (1.07–1.38)
Lag time (2 years)	408,028	501	0.12	407,848	523	0.13	1.05 (0.99–1.12)
Without compelling indications	253,448	472	0.19	252,315	580	0.23	1.20 (1.10–1.30)
As‐treated approach	408,491	686	0.17	408,491	913	0.22	1.18 (1.08–1.28)
Without propensity score matching	727,065	1783	0.25	492,558	1460	0.30	1.18 (1.10–1.27)

Abbreviations: ACEI, angiotensin‐converting enzyme inhibitor; ARB, angiotensin receptor blocker; cDDD, cumulative defined daily dose; CI, confidence interval; dCCB, dihydropyridine calcium channel blocker; HR, hazard ratio.

^a^ For sensitivity analysis, the model was adjusted for all confounders, which were used in the matching model in the main study, to account for potential imbalance between two groups.

### 3.4 Repeated Analysis: Comparing Non‐dCCB Use With ACEI or ARB Use

The results of the additional analyses comparing non‐dCCB use with ACEI or ARB use were presented in Tables S8–S13. Compared non‐dCCB use to ACEI or ARB use, no significant differences in the risk of kidney cancer were observed.

## 4 Discussion

To our knowledge, this is the first long‐term, large PS‐matched retrospective cohort study that investigated the risk of kidney cancer across the three most commonly used antihypertensive medications in hypertensive population using head‐to‐head comparison design. We observed that dCCB use was associated with an increased risk of kidney cancer compared to ACEI or ARB use. In addition, ARB use was associated with a higher risk of kidney cancer compared to ACEI use. In subgroup analyses, similar associations were generally observed, whereas null associations were shown in those with renal failure or COPD. Robust results from the sensitivity analyses supported the main findings. In contrast, there was no significant association of non‐dCCB use with kidney cancer risk compared to ACEI or ARB use.

Although no previous cohort study has directly compared the risk of kidney cancer between different classes of antihypertensive medications using PS matching, several case–control studies have supported our findings. In one case–control study, dCCB use was associated with a 30% higher risk of renal cell carcinoma (RCC) (odds ratio [OR] 1.32, 95% CI 1.05–1.67) compared to ARB use. Compared to ACEI use, dCCB use showed a trend of an increased risk of RCC (OR 1.21, 95% CI 0.95–1.53) [13]. In another case–control study conducted in a hypertensive population, CCB, but not differentiating dCCB and non‐dCCB, use was associated with a two‐fold higher risk of kidney cancer compared with non‐CCB, combining ACEI, BB, and DU [24]. Although they applied an active comparator design, two major limitations exist in these studies. First, due to the exposure information collected based on the outcome occurred in a case–control study design, there may be a lack of representative exposure distributions, and this leads to potential selection bias and lack of temporal associations. Second, since no information regarding the initiation of exposure to the medication was identified, indication bias and prevalent user bias could influence their results. When compared to non‐use, various classes of antihypertensive medication use were differently associated with 10%–50% increased risks of kidney cancer [25]. Although the results supported our findings that CCB had the higher risk of kidney cancer among different classes, it is important to note that comparing non‐use is susceptible to introduce indication bias. Contrary to these studies, the current PS‐matched cohort study provides more reliable relative risks with reduced bias. In this study, dCCB use was found to be associated with the highest risk of kidney cancer compared to ACEI and ARB use. This study contributes to the existing literature and clinical practice by providing evidence that medication selection may be a modifiable factor in minimizing a higher risk of kidney cancer in a hypertensive population, who need pharmacotherapy but are already at a high risk for kidney cancer, while simultaneously providing optimal hypertension care in accordance with the current guidelines.

Although similar results were observed across the pre‐defined subgroups, the associations were different according to the presence of renal failure or COPD. In patients with renal failure or COPD, no differences in the risk of kidney cancer were observed when comparing dCCB use to ACEI or ARB use. Additional risk from selecting antihypertensive medication use may be likely small in hypertensive patients with renal failure or COPD, who are already susceptible to a high risk of kidney cancer. Renal failure has been associated with a three‐ to ten‐fold increased risk of kidney cancer compared to the general population [26, 27, 28]. COPD history was used as a proxy of smoking [29], which is a strong risk factor for kidney cancer [4, 30, 31, 32].

Potential mechanisms underpinning carcinogenesis of antihypertensive medications are available, while stronger evidence exists for CCB compared to ACEI or ARB. Several studies found that CCB may allow for an accumulation of damaged cells such as potentially malignant genetic variants and trigger calcium‐mediated cell differentiation and apoptosis [10, 33, 34]. On the other hand, ACEI or ARB has conflicting evidence of both carcinogenic and anti‐cancer effects. ACEI may contribute to developing kidney cancer via increasing bradykinin levels [35], whereas ARB may promote cellular proliferation or angiogenesis and lead to angiotensin‐mediated tumor progression [11]. In contrast, ACEI or ARB may down‐regulate vascular endothelial growth factor‐mediated angiogenesis through angiotensin II and angiotensin receptors, which could potentially have anti‐cancer effects for skin, urogenital, or breast cancers [36, 37, 38]. Since our study compared dCCB and ACEI or ARB directly, we were unable to rule out the possibility that the observed increase in kidney cancer risk with dCCB may partly result from ACEI or ARB reducing the risk, rather than solely from dCCB increasing it. Therefore, we suggest future studies to clarify the potential carcinogenic effects of CCB compared to the protective effects of ACEI or ARB on kidney cancer risk. In fact, the current study found differences in kidney cancer risk when comparing dCCB with ACEI or ARB, while we observed null associations for non‐dCCB. As limited experimental studies that distinguish between dCCB and non‐dCCB are available, further research is warranted to explore their potentially distinct carcinogenic mechanisms.

For patients with hypertension, who are already at‐risk of developing kidney cancer but need to control their blood pressure using antihypertensive medications, every effort to provide evidence‐based prevention strategies for kidney cancer is necessary. Given their life‐long use and various mechanisms of action, the choice of antihypertensive medications might influence the risk of kidney cancer differently. Until now, potential carcinogenic mechanisms of dCCB have been less extensively investigated. Our findings emphasize the need for further investigation into potential biological links between dCCB use and kidney cancer development.

Our study has several strengths. First, our study with a long‐term, large analytic cohort allowed us to reduce selection and recall bias and apply systematic approaches to assess the robustness using several subgroup and sensitivity analyses. Second, our PS‐matched cohort reduced indication bias by balancing characteristics between study groups, while the new user, active comparator design minimized prevalent user bias by identifying treatment initiators, unlike previous case–control studies. Third, comprehensive diagnosis and prescription data helped us estimate cumulative exposure to each medication over the study period. Also, we were able to account for hypertensive complications or other potential compelling indications of medications and adherence to the medications. We were further able to account for other medications, such as analgesics, anti‐diabetic agents, or lipid‐lowering agents, which might be associated with a risk of kidney cancer [12, 21]. Our findings suggest that given that antihypertensive drugs are used over long‐term periods, the risk of kidney cancer should be carefully considered when selecting the primary agent for hypertension treatment.

Several limitations should also be noted. First, due to the nature of observational studies using claims data, we were unable to confirm whether patients took the prescribed medications. To mitigate this concern, we restricted the study to patients who had been prescribed antihypertensive medications for more than 6 months with an adherent use, indicating their regular use. In addition, we were unable to investigate the associations based on lifestyle factors including smoking status or body mass index, which are known risk factors for kidney cancer. Also, there could be unmeasured confounders related to severity of hypertension, potentially influencing the impacts of antihypertensive medications on kidney cancer. Indeed, it is impossible to separate the influence of high blood pressure or hypertension severity from the effects of antihypertensive medications on kidney cancer. More severe hypertension could be associated with higher cumulative dose and longer use of medications, as well as kidney cancer risk. However, in this PS‐matched study with an active comparator, this potential confounding effect is largely nondifferential across the study groups and usually biases the results toward null. Robust findings from dose–response relationship, along with different subgroup and sensitivity analyses including only longer users, suggest a possible association of different antihypertensive medication use with an elevated risk of kidney cancer, independent of hypertension. Second, we were unable to fully reflect on real‐world settings, which often involve more complex practices such as switching to another medication class or using combination therapies for patients with uncontrolled or severe hypertensive or for those with different indications. Although it may seem unrealistic to conduct observational studies to gather evidence on this matter, our findings suggest that dCCB have potential carcinogenic effects for kidney cancer that differ from those of other classes. Further studies are needed to explore the associations between different combination therapies and kidney cancer risk. Lastly, our study patients from the MarketScan Databases may remain relatively stable in their enrollment in health insurance in comparison to the general population [17]. Accordingly, the population may not fully represent individuals with a low socioeconomic status.

In conclusion, we found that dCCB use was associated with an increased risk of kidney cancer compared to ACEI or ARB use in a hypertensive population. Given their long‐term use and the fact that patients with hypertension are already at‐risk of developing kidney cancer, the choice of antihypertensive medications may be one of modifiable factors in minimizing the potential risk of kidney cancer while also controlling blood pressure and preventing cardiovascular diseases.

## Author Contributions

**Minji Jung:** conceptualization (lead), data curation (lead), formal analysis (lead), investigation (lead), methodology (equal), software (lead), visualization (lead), writing – original draft (lead), writing – review and editing (lead). **Shufeng Li:** methodology (supporting). **Zhengyi Deng:** writing – review and editing (supporting). **Jinhui Li:** writing – review and editing (supporting). **Mingyi Li:** writing – review and editing (supporting). **Satvir Basran:** writing – review and editing (supporting). **Marvin E. Langston:** conceptualization (equal), investigation (equal), methodology (equal), project administration (lead), supervision (lead), validation (equal), writing – review and editing (lead). **Benjamin I. Chung:** conceptualization (equal), project administration (lead), supervision (lead), writing – review and editing (supporting).

## Conflicts of Interest

The authors declare no conflicts of interest.

## Supporting information


Data S1.

## Data Availability

The study used the Merative MarketScan Commercial and Medicare Databases. The authors cannot legally distribute these data, but details on data access can be found here: https://www.merative.com/content/dam/merative/documents/brief/marketscan‐explainer‐general.pdf.
