# Supplementary material for: Calcium Channel Blocker Versus Renin–Angiotensin System Inhibitor in Risk of Kidney Cancer Among Patients With Hypertension: A Propensity Score‐Matched Cohort Study
Source: Cancer Med. 2024 Nov 16;13(22):e70429. doi: 10.1002/cam4.70429 (PMC11568363; doi:10.1002/cam4.70429)
Supplement: Supplementary file 1 — Data S1. [file CAM4-13-e70429-s001.docx]

Table S1. Definition of comorbidities and study outcome using ICD-9 and ICD-10 codes

Table S2. Lists of antihypertensive drugs as generic drug names

Table S3. Results of subgroup analyses for kidney cancer risk according to potential covariates compared ARB to ACEI

Table S4. Results of subgroup analyses for kidney cancer risk according to potential covariates compared dCCB to ACEI

Table S5. Results of subgroup analyses for kidney cancer risk according to potential covariates compared dCCB to ARB

Table S6. Results of subgroup analyses for kidney cancer risk according to previous use of other AHTN with the index AHTN

Table S7. Results of exposure to the index AHTN including ACEI, ARB, and dCCB with the risk of kidney cancer

Table S8. Baseline characteristics of non-dCCB versus ACEI and ARB

Table S9. Risks of kidney cancer associated with non-dCCB versus ACEI and ARB

Table S10. Results of subgroup analyses for kidney cancer risk compared non-dCCB to ACEI

Table S11. Results of subgroup analyses for kidney cancer risk compared non-dCCB to ARB

Table S12. Results of exposure to the other AHTN including ACEI, ARB, and non-dCCB with the risk of kidney cancer

Table S13. Results of sensitivity analyses for kidney cancer risk compared non-dCCB to ACEI and ARB

Figure S1. Flowchart for the selection of study participants of new users of ACEI, ARB, and CCB

Table S1. Definition of comorbidities and study outcome using ICD-9 and ICD-10 codes

| Comorbidities | ICD-9-CM Codes | ICD-10-CM Codes |
| --- | --- | --- |
| Atrial fibrillation | 427.3 | I48.x |
| Hypertension | 401.x-405.x | I10.x–I15.x |
| Dyslipidemia | 272.x | E78.x |
| Angina | 413.x | I20.x, I25.11, I25.7x |
| Chronic obstructive pulmonary disease | 491.x-492.x, 493.2, 496.x | J41.x-J44.x |
| Renal failure, dialysis, or renal transplantation | 403.01, 403.11, 403.91, 404.02, 404.12, 404.92, 585.5, 585.6, 586.x, V42.0, V45.11, V56.1, V56.2, | I12.0, I13.11, N18.5, N18.6, N19.x, Z49.0, Z94.0, Z99.2 |
| Ischemic stroke/transient ischemic attack | 433.x-436.x | I63.x, G45.x |
| Hemorrhagic stroke | 430.x-432.x, 852.x-853.x | I60.x-I62.x |
| **CCI** | ICD-9-CM Codes | ICD-10-CM Codes |
|  | Weight=1 |  |
| Cerebrovascular disease | 362.34, 430.x, 431.x, 432.x, 433.x, 434.x, 435.x, 436.x, 437.x, 438.x | G45.x, G46.x, H34.0, I60.x-I69.x |
| Congestive heart failure | 398.91, 402.x1, 404.x3, 428.x | I50.x |
| Chronic pulmonary disease | 490.x, 491.x, 492.x, 493.x, 494.x, 495.x, 496.x, 500.x, 501.x, 502.x, 503.x, 504.x, 505.x, 506.4, 508.1, 508.8 | I27.8, I27.9, J40.x–J47.x, J60.x–J67.x, J68.4, J70.1, J70.3 |
| Dementia | 290.0-290.4, 294.1, 294.2, 330.8, 330.9, 331.0, 331.1, 331.2, 331.6, 331.7, 331.82, 331.83, 331.89, 331.9, 334.4, 336.2, 349.89 | F01.x–F03.x, G30.x–G32.x |
| Diabetes without chronic complication | 250.0 | E10.0, E10.1, E10.6, E10.8, E10.9, E11.0, E11.1, E11.6, E11.8, E11.9, E13.0, E13.1, E13.6, E13.8, E13.9 |
| Mild liver disease | 070.22, 070.23, 070.32, 070.33, 070.44, 070.54, 070.6, 070.9, 570.x, 571.x, 573.3, 573.4, 573.8,  573.9, V42.7 | B18.x, K70.0-K70.3, K70.9, K71.3-K71.5, K71.7, K73.x, K74.x, K76.0, K76.2-K76.4, K76.8, K76.9, Z94.4 |
| Mild or moderate renal disease | 403.00, 403.10, 403.90, 404.00, 404.01, 404.10, 404.11, 404.90, 404.91, 582.x, 583.x, 585.1, 585.2,  585.3, 585.4, 585.9, V42.0 | I12.9, I13.0, I13.10, N03.x, N05.x, N18.1, N18.2, N18.3, N18.4, N18.9, Z94.0 |
| Myocardial infarction | 410, 412 | I21.x, I22.x, I25.2 |
| Peripheral vascular disease | 093.0, 437.3, 440.x, 441.x, 443.1, 443.2x, 443.8x 443.9, 447.1, 557.1, 557.9, V43.4 | I70.x, I71.x, I73.1, I73.8, I73.9, I77.1, I79.0, I79.2, K55.1, K55.8, K55.9, Z95.8, Z95.9 |
| Peptic ulcer disease | 531.x, 532.x, 533.x, 534.x | K25.x–K28.x, |
| Rheumatologic disease | 446.5, 710.0, 710.1, 710.2, 710.3, 710.4, 714.0, 714.1, 714.2, 714.8x, 725.x | M05.x, M06.x, M32.x–M34.x M31.5M35.1, M35.3, M36.0 |
|  | **Weight=2** |  |
| Diabetes with chronic complication | 250.1-250.9, 357.2, 362.0, 366.41 | E10.2–E10.5, E11.2–E11.5, E13.2–E13.5, E14.x |
| Hemiplegia or paraplegia | 334.1, 342.x, 343.x, 344.x | G04.1, G11.4, G80.1, G80.2, G81.x, G82.x, G83.x |
| Any malignancy, including leukemia and lymphoma | 14x.x, 15x.x, 16x.x, 170.x, 171.x, 172.x, 174.x, 175.x, 176.x, 179.x, 18x.x, 190.x, 191.x, 192.x, 193.x, 194.x, 195.x, 199.1, 200.x, 201.x, 202.x, 203.x, 204.x, 205.x, 206.x, 207.x, 208.x, 238.6 | C00.x–C26.x, C30.x–C34.x, C37.x–C41.x, C43.x, C45.x–C58.x, C60.x–C76.x, C81.x–C85.x, C88.x, C90.x–C97.x, |
|  | **Weight=3** |  |
| Moderate or severe liver disease | 456.0, 456.1, 456.2x, 572.2, 572.3, 572.4, 572.8 | I85.0x, I86.4, K70.4x, K71.1x, K72.1x, K72.9x, K76.5, K76.6, K76.7 |
| Severe renal disease | 403.01, 403.11, 403.91, 404.02, 404.03, 404.12, 404.13, 404.92, 404.93, 585.5, 585.6, 586.x, 588.0, V42.0, V45.11, V45.12, V56.0, V56.1, V56.2, V56.31, V56.32, V56.8 | I12.0, I13.x, N03.2-N03.7, N05.2-N05.7, N18.5, N18.6, N19.x, N25.0, Z49.0-Z49.2, Z94.0, Z99.2 |
|  | **Weight=6** |  |
| HIV/AIDS | 042.x | B20.x |
| Metastatic solid tumor | 196.x-199.0 | C77.x–C80.0, C80.2 |
| Abbreviations. CCI, Charlson Comorbidity Index; HIV/AIDS, human immunodeficiency virus infection and acquired immunodeficiency syndrome; ICD, International Classification of Diseases | | |

Table S2. Lists of antihypertensive drugs as generic drug names

| **Class** | **Active ingredients** |
| --- | --- |
| ACEI | Benazepril, Captopril, Enalapril, Fosinopril, Lisinopril, Moexipril, Perindopril, Quinapril, Ramipril, Trandolapril |
| ARB | Candesartan, Eprosartan, Irbesartan, Losartan, Olmesartan, Telmisartan, Valsartan |
| dCCB | Amlodipine, Felodipine, Isradipine, Nicardipine, Nifedipine, Nimodipine, Nisoldipine |
| Non-dCCB | Diltiazem, Verapamil |
| BB | Acebutolol, Atenolol, Betaxolol, Bisoprolol, Carvedilol, Metoprolol, Nadolol, Nebivolol, Penbutolol, Pindolol, Propranolol |
| DU | Amiloride, Bendroflumethiazide, Bumetanide, Chlorothiazide, Chlorthalidone, Eplerenone, Furosemide, Hydrochlorothiazide, Indapamide, Methazolamide, Methyclothiazide, Metolazone, Spironolactone, Torsemide, Triamterene |
| Abbreviations: ACEI, angiotensin-converting enzyme inhibitor; ARB, angiotensin receptor blocker; BB, beta-blocker; dCCB, dihydropyridine calcium channel blocker; DU, diuretic | |

Table S3. Results of subgroup analyses for kidney cancer risk according to potential covariates compared ARB to ACEI

|  | ACEI | | | ARB | | | ARB vs ACEI |  |
| --- | --- | --- | --- | --- | --- | --- | --- | --- |
|  | N=640,671 | | | N=640,671 | | |  |  |
| Subgroups † | N | Events, N | (%) | N | Events, N | (%) | Adjusted HR (95% CI) ‡ | p for difference |
| Age |  |  |  |  |  |  |  |  |
| < 50 years | 144,101 | 178 | 0.12% | 135,786 | 166 | 0.12% | 1.07 (0.87-1.33) |  |
| 50-60 years | 217,283 | 456 | 0.21% | 216,302 | 510 | 0.24% | 1.18 (1.04-1.34) | 0.387 |
| 60-70 years | 180,868 | 506 | 0.28% | 186,666 | 478 | 0.26% | 1.02 (0.89-1.14) |  |
| ≥ 70 years | 98,419 | 411 | 0.42% | 101,917 | 424 | 0.42% | 1.11 (0.96-1.25) |  |
| Sex |  |  |  |  |  |  |  |  |
| Male | 323,866 | 1,007 | 0.31% | 319,898 | 971 | 0.30% | 1.05 (0.97-1.15) |  |
| Female | 316,805 | 544 | 0.17% | 320,773 | 607 | 0.19% | 1.18 (1.05-1.33) | 0.153 |
| Myocardial infarction |  |  |  |  |  |  |  |  |
| No | 621,799 | 1,483 | 0.24% | 621,818 | 1,519 | 0.24% | 1.11 (1.03-1.19) |  |
| Yes | 18,872 | 68 | 0.36% | 18,853 | 59 | 0.31% | 0.97 (0.68-1.37) | 0.455 |
| Ischemic stroke/TIA |  |  |  |  |  |  |  |  |
| No | 599,529 | 1,400 | 0.23% | 599,163 | 1,447 | 0.24% | 1.12 (1.04-1.20) |  |
| Yes | 41,142 | 151 | 0.37% | 41,508 | 131 | 0.32% | 0.94 (0.74-1.19) | 0.162 |
| DM without complications |  |  |  |  |  |  |  |  |
| No | 483,536 | 1,025 | 0.21% | 478,319 | 1,065 | 0.22% | 1.14 (1.05-1.24) |  |
| Yes | 157,135 | 526 | 0.33% | 162,352 | 513 | 0.32% | 1.02 (0.90-1.15) | 0.121 |
| DM with complications |  |  |  |  |  |  |  |  |
| No | 596,061 | 1,401 | 0.24% | 592,870 | 1,399 | 0.24% | 1.09 (1.01-1.18) |  |
| Yes | 44,610 | 150 | 0.34% | 47,801 | 179 | 0.37% | 1.17 (0.94-1.46) | 0.535 |
| Heart failure |  |  |  |  |  |  |  |  |
| No | 609,562 | 1,442 | 0.24% | 609,614 | 1,462 | 0.24% | 1.09 (1.02-1.18) |  |
| Yes | 31,109 | 109 | 0.35% | 31,057 | 116 | 0.37% | 1.14 (0.91-1.54) | 0.564 |
| Atrial fibrillation |  |  |  |  |  |  |  |  |
| No | 608,835 | 1,431 | 0.24% | 608,503 | 1,448 | 0.24% | 1.09 (1.01-1.17) |  |
| Yes | 31,836 | 120 | 0.38% | 32,168 | 130 | 0.40% | 1.20 (0.93-1.54) | 0.481 |
| Dyslipidemia |  |  |  |  |  |  |  |  |
| No | 266,109 | 579 | 0.22% | 262,377 | 610 | 0.23% | 1.15 (1.03-1.29) |  |
| Yes | 374,562 | 972 | 0.26% | 378,294 | 968 | 0.26% | 1.07 (0.98-1.17) | 0.291 |
| Peripheral vascular disease |  |  |  |  |  |  |  |  |
| No | 598,504 | 1,398 | 0.23% | 597,049 | 1,439 | 0.24% | 1.11 (1.03-1.20) |  |
| Yes | 42,167 | 153 | 0.36% | 43,622 | 139 | 0.32% | 0.98 (0.78-1.23) | 0.290 |
| Chronic kidney disease |  |  |  |  |  |  |  |  |
| No | 611,968 | 1,393 | 0.23% | 609,490 | 1,418 | 0.23% | 1.11 (1.03-1.20) |  |
| Yes | 28,703 | 158 | 0.55% | 31,181 | 160 | 0.51% | 1.01 (0.81-1.26) | 0.438 |
| Renal failure |  |  |  |  |  |  |  |  |
| No | 634,430 | 1,501 | 0.24% | 634,030 | 1,524 | 0.24% | 1.10 (1.02-1.18) |  |
| Yes | 6,241 | 50 | 0.80% | 6,641 | 54 | 0.81% | 1.13 (0.77-1.66) | 0.807 |
| COPD |  |  |  |  |  |  |  |  |
| No | 601,975 | 1,409 | 0.23% | 601,597 | 1,439 | 0.24% | 1.10 (1.02-1.18) |  |
| Yes | 38,696 | 142 | 0.37% | 39,074 | 139 | 0.36% | 1.06 (0.88-1.35) | 0.744 |
| Abbreviations: ACEI, angiotensin-converting enzyme inhibitor; ARB, angiotensin receptor blocker; CI, confidence interval; COPD, chronic obstructive pulmonary disease; dCCB, dihydropyridine calcium channel blocker; HR, hazard ratio; TIA, transient ischemic attack  † Due to the small number (<11) of kidney cancer incidence in subgroups of hemorrhagic stroke and angina, we were unable to present the data.  ‡ The HR was adjusted for Charlson comorbidity index. | | | | | | | | |

Table S4. Results of subgroup analyses for kidney cancer risk according to potential covariates compared dCCB to ACEI

|  | ACEI | | | dCCB | | | dCCB vs ACEI |  |
| --- | --- | --- | --- | --- | --- | --- | --- | --- |
|  | N=373,601 | | | N=373,601 | | |  |  |
| Subgroups † | N | Events, N | (%) | N | Events, N | (%) | Adjusted HR (95% CI) ‡ | p for difference |
| Age |  |  |  |  |  |  |  |  |
| < 50 years | 81,955 | 106 | 0.13% | 83,009 | 118 | 0.14% | 1.19 (0.91-1.54) |  |
| 50-60 years | 119,266 | 240 | 0.20% | 118,960 | 335 | 0.28% | 1.46 (1.24-1.72) | 0.270 |
| 60-70 years | 105,326 | 279 | 0.26% | 104,625 | 337 | 0.32% | 1.28 (1.09-1.51) |  |
| ≥ 70 years | 67,054 | 269 | 0.40% | 67,007 | 301 | 0.45% | 1.21 (1.02-1.39) |  |
| Sex |  |  |  |  |  |  |  |  |
| Male | 190,213 | 591 | 0.31% | 192,239 | 730 | 0.38% | 1.28 (1.15-1.43) |  |
| Female | 183,388 | 303 | 0.17% | 181,362 | 361 | 0.20% | 1.29 (1.12-1.48) | 0.979 |
| Myocardial infarction |  |  |  |  |  |  |  |  |
| No | 362,177 | 858 | 0.24% | 362,092 | 1,034 | 0.29% | 1.27 (1.16-1.39) |  |
| Yes | 11,424 | 36 | 0.32% | 11,509 | 57 | 0.50% | 1.64 (1.08-2.49) | 0.240 |
| Ischemic stroke/TIA |  |  |  |  |  |  |  |  |
| No | 344,628 | 795 | 0.23% | 345,059 | 978 | 0.28% | 1.29 (1.18-1.42) |  |
| Yes | 28,973 | 99 | 0.34% | 28,542 | 113 | 0.40% | 1.20 (0.94-1.58) | 0.622 |
| DM without complications |  |  |  |  |  |  |  |  |
| No | 291,215 | 593 | 0.20% | 290,524 | 783 | 0.27% | 1.41 (1.12-1.57) |  |
| Yes | 82,386 | 301 | 0.37% | 83,077 | 308 | 0.37% | 1.06 (0.92-1.26) | 0.052 |
| DM with complications |  |  |  |  |  |  |  |  |
| No | 346,376 | 782 | 0.23% | 345,752 | 987 | 0.29% | 1.34 (1.22-1.47) |  |
| Yes | 27,225 | 112 | 0.41% | 27,849 | 104 | 0.37% | 1.05 (0.75-1.32) | 0.061 |
| Heart failure |  |  |  |  |  |  |  |  |
| No | 356,146 | 827 | 0.23% | 356,865 | 1,023 | 0.29% | 1.30 (1.19-1.42) |  |
| Yes | 17,455 | 67 | 0.38% | 16,736 | 68 | 0.41% | 1.08 (0.78-1.51) | 0.302 |
| Atrial fibrillation |  |  |  |  |  |  |  |  |
| No | 356,667 | 828 | 0.23% | 357,752 | 1,025 | 0.29% | 1.30 (1.18-1.42) |  |
| Yes | 16,934 | 66 | 0.39% | 15,849 | 66 | 0.42% | 1.09 (0.77-1.53) | 0.323 |
| Dyslipidemia |  |  |  |  |  |  |  |  |
| No | 163,336 | 360 | 0.22% | 163,735 | 457 | 0.28% | 1.32 (1.15-1.52) |  |
| Yes | 210,265 | 534 | 0.25% | 209,866 | 634 | 0.30% | 1.27 (1.12-1.41) | 0.556 |
| Peripheral vascular disease |  |  |  |  |  |  |  |  |
| No | 345,427 | 791 | 0.23% | 345,393 | 998 | 0.29% | 1.33 (1.21-1.46) |  |
| Yes | 28,174 | 103 | 0.37% | 28,208 | 93 | 0.33% | 1.15 (0.86-1.34) | 0.067 |
| Chronic kidney disease |  |  |  |  |  |  |  |  |
| No | 352,605 | 780 | 0.22% | 350,577 | 964 | 0.27% | 1.32 (1.20-1.45) |  |
| Yes | 20,996 | 114 | 0.54% | 23,024 | 127 | 0.55% | 1.08 (0.84-1.36) | 0.115 |
| Renal failure |  |  |  |  |  |  |  |  |
| No | 369,040 | 852 | 0.23% | 368,425 | 1,058 | 0.29% | 1.31 (1.20-1.43) |  |
| Yes | 4,561 | 42 | 0.92% | 5,176 | 33 | 0.64% | 0.92 (0.49-1.27) | 0.042 |
| COPD |  |  |  |  |  |  |  |  |
| No | 349,365 | 800 | 0.23% | 350,404 | 999 | 0.29% | 1.31 (1.19-1.43) |  |
| Yes | 24,236 | 94 | 0.39% | 23,197 | 92 | 0.40% | 0.95 (0.83-1.22) | 0.035 |
| Abbreviations: ACEI, angiotensin-converting enzyme inhibitor; ARB, angiotensin receptor blocker; CI, confidence interval; COPD, chronic obstructive pulmonary disease; dCCB, dihydropyridine calcium channel blocker; HR, hazard ratio; TIA, transient ischemic attack  † Due to the small number (<11) of kidney cancer incidence in subgroups of hemorrhagic stroke and angina, we were unable to present the data.  ‡ The HR was adjusted for Charlson comorbidity index. | | | | | | | | |

Table S5. Results of subgroup analyses for kidney cancer risk according to potential covariates compared dCCB to ARB

|  | ARB | | | dCCB | | | dCCB vs ARB |  |
| --- | --- | --- | --- | --- | --- | --- | --- | --- |
|  | N=408,491 | | | N=408,491 | | |  |  |
| Subgroups † | N | Events, N | (%) | N | Events, N | (%) | Adjusted HR (95% CI) ‡ | p for difference |
| Age |  |  |  |  |  |  |  |  |
| < 50 years | 85,963 | 96 | 0.11% | 90,333 | 121 | 0.13% | 1.18 (0.90-1.55) |  |
| 50-60 years | 131,206 | 292 | 0.22% | 128,997 | 357 | 0.28% | 1.21 (1.04-1.42) | 0.858 |
| 60-70 years | 117,934 | 291 | 0.25% | 114,677 | 354 | 0.31% | 1.19 (1.02-1.38) |  |
| ≥ 70 years | 73,388 | 285 | 0.39% | 74,484 | 334 | 0.45% | 1.11 (0.95-1.28) |  |
| Sex |  |  |  |  |  |  |  |  |
| Male | 206,499 | 605 | 0.29% | 210,215 | 745 | 0.35% | 1.17 (1.05-1.31) |  |
| Female | 201,992 | 359 | 0.18% | 198,276 | 421 | 0.21% | 1.16 (1.02-1.34) | 0.928 |
| Myocardial infarction |  |  |  |  |  |  |  |  |
| No | 395,342 | 924 | 0.23% | 395,469 | 1,111 | 0.28% | 1.16 (1.06-1.27) |  |
| Yes | 13,149 | 40 | 0.30% | 13,022 | 55 | 0.42% | 1.32 (0.88-1.98) | 0.559 |
| Ischemic stroke/TIA |  |  |  |  |  |  |  |  |
| No | 378,374 | 880 | 0.23% | 376,942 | 1,054 | 0.28% | 1.16 (1.06-1.27) |  |
| Yes | 30,117 | 84 | 0.28% | 31,549 | 112 | 0.36% | 1.26 (0.95-1.67) | 0.585 |
| DM without complications |  |  |  |  |  |  |  |  |
| No | 314,767 | 664 | 0.21% | 312,905 | 809 | 0.26% | 1.18 (1.07-1.31) |  |
| Yes | 93,724 | 300 | 0.32% | 95,586 | 357 | 0.37% | 1.13 (0.98-1.32) | 0.646 |
| DM with complications |  |  |  |  |  |  |  |  |
| No | 375,958 | 846 | 0.23% | 375,380 | 1,035 | 0.28% | 1.18 (1.08-1.29) |  |
| Yes | 32,533 | 118 | 0.36% | 33,111 | 131 | 0.40% | 1.08 (0.84-1.38) | 0.495 |
| Heart failure |  |  |  |  |  |  |  |  |
| No | 388,659 | 895 | 0.23% | 388,926 | 1,099 | 0.28% | 1.19 (1.09-1.30) |  |
| Yes | 19,832 | 69 | 0.35% | 19,565 | 67 | 0.34% | 0.92 (0.68-1.27) | 0.134 |
| Atrial fibrillation |  |  |  |  |  |  |  |  |
| No | 389,880 | 889 | 0.23% | 390,432 | 1,094 | 0.28% | 1.19 (1.11-1.31) |  |
| Yes | 18,611 | 75 | 0.40% | 18,059 | 72 | 0.40% | 0.91 (0.66-1.26) | 0.124 |
| Dyslipidemia |  |  |  |  |  |  |  |  |
| No | 169,842 | 371 | 0.22% | 174,666 | 467 | 0.27% | 1.18 (1.03-1.36) |  |
| Yes | 238,649 | 593 | 0.25% | 233,825 | 699 | 0.30% | 1.16 (1.05-1.29) | 0.801 |
| Peripheral vascular disease |  |  |  |  |  |  |  |  |
| No | 376,248 | 865 | 0.23% | 376,121 | 1,050 | 0.28% | 1.17 (1.07-1.29) |  |
| Yes | 32,243 | 99 | 0.31% | 32,370 | 116 | 0.36% | 1.11 (0.85-1.45) | 0.709 |
| Chronic kidney disease |  |  |  |  |  |  |  |  |
| No | 380,590 | 821 | 0.22% | 379,382 | 1,024 | 0.27% | 1.21 (1.10-1.33) |  |
| Yes | 27,901 | 143 | 0.51% | 29,109 | 142 | 0.49% | 0.94 (0.74-1.18) | 0.053 |
| Renal failure |  |  |  |  |  |  |  |  |
| No | 402,771 | 929 | 0.23% | 402,156 | 1,135 | 0.28% | 1.18 (1.08-1.29) |  |
| Yes | 5,720 | 35 | 0.61% | 6,335 | 31 | 0.49% | 0.78 (0.49-1.11) | 0.018 |
| COPD |  |  |  |  |  |  |  |  |
| No | 382,006 | 866 | 0.23% | 382,078 | 1,079 | 0.28% | 1.20 (1.10-1.31) |  |
| Yes | 26,485 | 98 | 0.37% | 26,413 | 87 | 0.33% | 0.87 (0.66-1.15) | 0.036 |
| Abbreviations: ACEI, angiotensin-converting enzyme inhibitor; ARB, angiotensin receptor blocker; CI, confidence interval; COPD, chronic obstructive pulmonary disease; dCCB, dihydropyridine calcium channel blocker; HR, hazard ratio; TIA, transient ischemic attack  † Due to the small number (<11) of kidney cancer incidence in subgroups of hemorrhagic stroke and angina, we were unable to present the data.  ‡ The HR was adjusted for Charlson comorbidity index. | | | | | | | | |

Table S6. Results of subgroup analyses for kidney cancer risk according to previous use of other AHTN with the index AHTN

|  | ACEI | | | | ARB | | | | ARB vs ACEI |
| --- | --- | --- | --- | --- | --- | --- | --- | --- | --- |
| Study cohort 1 | N=640,671 | | | | N=640,671 | | | |  |
|  | N | (%) | Event, N | (%) | N | (%) | Event, N | (%) | Adjusted HR  (95% CI) † |
| Previous use of BB |  |  |  |  |  |  |  |  |  |
| No | 556,020 | 86.8% | 1,319 | 0.2% | 559,428 | 87.3% | 1,364 | 0.2% | 1.10 (1.03-1.18) |
| Yes | 84,651 | 13.2% | 232 | 0.3% | 81,243 | 12.7% | 214 | 0.3% | 1.14 (0.94-1.36) |
| p for difference |  |  |  |  |  |  |  |  | 0.715 |
| Previous use of DU |  |  |  |  |  |  |  |  |  |
| No | 536,885 | 83.8% | 1,234 | 0.2% | 540,989 | 84.4% | 1,293 | 0.2% | 1.11 (1.03-1.21) |
| Yes | 103,786 | 16.2% | 317 | 0.3% | 99,682 | 15.6% | 285 | 0.3% | 1.05 (0.90-1.22) |
| p for difference |  |  |  |  |  |  |  |  | 0.451 |
| Previous use of loop DU |  |  |  |  |  |  |  |  |  |
| No | 615,052 | 96.0% | 1,455 | 0.2% | 615,373 | 96.1% | 1,496 | 0.2% | 1.11 (1.03-1.19) |
| Yes | 25,619 | 4.0% | 96 | 0.4% | 25,298 | 3.9% | 82 | 0.3% | 0.97 (0.72-1.30) |
| p for difference |  |  |  |  |  |  |  |  | 0.440 |
| Previous use of K^+^ sparing DU |  |  |  |  |  |  |  |  |  |
| No | 618,317 | 96.5% | 1,478 | 0.2% | 619,479 | 96.7% | 1,518 | 0.2% | 1.11 (1.03-1.19) |
| Yes | 22,354 | 3.5% | 73 | 0.3% | 21,192 | 3.3% | 60 | 0.3% | 0.98 (0.70-1.39) |
| p for difference |  |  |  |  |  |  |  |  | 0.512 |
| Previous use of thiazide DU |  |  |  |  |  |  |  |  |  |
| No | 577,084 | 90.1% | 1,377 | 0.2% | 579,872 | 90.5% | 1,408 | 0.2% | 1.10 (1.02-1.18) |
| Yes | 63,587 | 9.9% | 174 | 0.3% | 60,799 | 9.5% | 170 | 0.3% | 1.12 (0.90-1.38) |
| p for difference |  |  |  |  |  |  |  |  | 0.840 |
|  | ACEI | | | | dCCB | | | | dCCB vs ACEI |
| Study cohort 2 | N=373,601 | | | | N=373,601 | | | |  |
|  | N | (%) | Event, N | (%) | N | (%) | Event, N | (%) | Adjusted HR  (95% CI) † |
| Previous use of BB |  |  |  |  |  |  |  |  |  |
| No | 318,413 | 85.2% | 757 | 0.2% | 321,271 | 86.0% | 916 | 0.3% | 1.26 (1.14-1.39) |
| Yes | 55,188 | 14.8% | 137 | 0.2% | 52,330 | 14.0% | 175 | 0.3% | 1.42 (1.15-1.78) |
| p for difference |  |  |  |  |  |  |  |  | 0.320 |
| Previous use of DU |  |  |  |  |  |  |  |  |  |
| No | 310,514 | 83.1% | 730 | 0.2% | 314,444 | 84.2% | 914 | 0.3% | 1.35 (1.24-1.43) |
| Yes | 63,087 | 16.9% | 164 | 0.3% | 59,157 | 15.8% | 177 | 0.3% | 1.13 (1.02-1.38) |
| p for difference |  |  |  |  |  |  |  |  | 0.105 |
| Previous use of loop DU |  |  |  |  |  |  |  |  |  |
| No | 358,665 | 96.0% | 845 | 0.2% | 362,300 | 97.0% | 1,051 | 0.3% | 1.31 (1.19-1.43) |
| Yes | 14,936 | 4.0% | 69 | 0.5% | 11,301 | 3.0% | 40 | 0.4% | 0.92 (0.64-1.34) |
| p for difference |  |  |  |  |  |  |  |  | 0.076 |
| Previous use of K^+^ sparing DU |  |  |  |  |  |  |  |  |  |
| No | 359,558 | 96.2% | 850 | 0.2% | 361,265 | 96.7% | 1,049 | 0.3% | 1.29 (1.18-1.41) |
| Yes | 14,043 | 3.8% | 44 | 0.3% | 12,336 | 3.3% | 42 | 0.3% | 1.17 (0.82-1.78) |
| p for difference |  |  |  |  |  |  |  |  | 0.670 |
| Previous use of thiazide DU |  |  |  |  |  |  |  |  |  |
| No | 335,356 | 89.8% | 791 | 0.2% | 334,077 | 89.4% | 982 | 0.3% | 1.32 (1.21-1.45) |
| Yes | 38,245 | 10.2% | 103 | 0.3% | 39,524 | 10.6% | 109 | 0.3% | 1.04 (0.83-1.37) |
| p for difference |  |  |  |  |  |  |  |  | 0.111 |
|  | ARB | | | | dCCB | | | | dCCB vs ARB |
| Study cohort 3 | N=408,491 | | | | N=408,491 | | | |  |
|  | N | (%) | Event, N | (%) | N | (%) | Event, N | (%) | Adjusted HR  (95% CI) † |
| Previous use of BB |  |  |  |  |  |  |  |  |  |
| No | 353,007 | \| 86.4% \| \| --- \| | 836 | 0.2% | 350,407 | 85.8% | 986 | 0.3% | 1.16 (1.06-1.27) |
| Yes | 55,484 | 13.6% | 128 | 0.2% | 58,084 | 14.2% | 180 | 0.3% | 1.24 (0.99-1.56) |
| p for difference |  |  |  |  |  |  |  |  | 0.580 |
| Previous use of DU |  |  |  |  |  |  |  |  |  |
| No | 335,947 | 82.2% | 753 | 0.2% | 340,947 | 83.5% | 965 | 0.3% | 1.23 (1.11-1.35) |
| Yes | 72,544 | 17.8% | 211 | 0.3% | 67,544 | 16.5% | 201 | 0.3% | 1.02 (0.89-1.26) |
| p for difference |  |  |  |  |  |  |  |  | 0.105 |
| Previous use of loop DU |  |  |  |  |  |  |  |  |  |
| No | 390,661 | 95.6% | 898 | 0.2% | 394,654 | 96.6% | 1,122 | 0.3% | 1.19 (1.09-1.30) |
| Yes | 17,830 | 4.4% | 66 | 0.4% | 13,837 | 3.4% | 44 | 0.3% | 0.89 (0.62-1.21) |
| p for difference |  |  |  |  |  |  |  |  | 0.066 |
| Previous use of K^+^ sparing DU |  |  |  |  |  |  |  |  |  |
| No | 393,284 | 96.3% | 918 | 0.2% | 395,187 | 96.7% | 1,118 | 0.3% | 1.17 (1.07-1.28) |
| Yes | 15,207 | 3.7% | 46 | 0.3% | 13,304 | 3.3% | 48 | 0.4% | 1.13 (0.80-1.68) |
| p for difference |  |  |  |  |  |  |  |  | 0.836 |
| Previous use of thiazide DU |  |  |  |  |  |  |  |  |  |
| No | 364,034 | 89.1% | 841 | 0.2% | 363,284 | 88.9% | 1,042 | 0.3% | 1.21 (1.10-1.32) |
| Yes | 44,457 | 10.9% | 123 | 0.3% | 45,207 | 11.1% | 124 | 0.3% | 0.98 (0.72-1.24) |
| p for difference |  |  |  |  |  |  |  |  | 0.054 |
| Abbreviations. ACEI, angiotensin-converting enzyme inhibitor; ARB, angiotensin receptor blocker; BB, beta-blocker; CI, confidence interval; DU, diuretic; HR, hazard ratio  † The HR was adjusted for Charlson comorbidity index. | | | | | | | | | |

Table S7. Results of exposure to the index AHTN including ACEI, ARB, and dCCB with the risk of kidney cancer

| Study cohort 1 | ACEI | | | | | | ARB | | | | | | ARB vs ACEI |
| --- | --- | --- | --- | --- | --- | --- | --- | --- | --- | --- | --- | --- | --- |
|  | N=640,671 | | | | | | N=640,671 | | | | | |  |
| Subgroups † | N | | Event, N | | (%) | | N | | Event, N | | | (%) | Adjusted HR (95% CI) ‡ |
| cDDD |  | |  | |  | |  | |  | | |  |  |
| < 500 cDDD | 232,952 | | 420 | | 0.18% | | 323,618 | | 566 | | | 0.17% | 1.05 (0.94-1.20) |
| 500-1500 cDDD | 227,213 | | 487 | | 0.21% | | 204,231 | | 548 | | | 0.27% | 1.09 (0.97-1.22) |
| 1500-3000 cDDD | 107,861 | | 317 | | 0.29% | | 78,779 | | 283 | | | 0.36% | 1.08 (0.92-1.26) |
| ≥ 3000 cDDD | 72,645 | | 327 | | 0.45% | | 34,043 | | 181 | | | 0.53% | 1.16 (0.97-1.39) |
| Duration |  | |  | |  | |  | |  | | |  |  |
| < 1 year | 217,000 | | 375 | | 0.17% | | 205,225 | | 291 | | | 0.14% | 1.07 (0.92-1.25) |
| 1-3 years | 260,093 | | 574 | | 0.22% | | 271,178 | | 653 | | | 0.24% | 1.14 (0.93-1.28) |
| 3-5 years | 98,901 | | 313 | | 0.32% | | 110,395 | | 381 | | | 0.35% | 1.09 (0.93-1.26) |
| 5-7 years | 40,712 | | 170 | | 0.42% | | 37,275 | | 160 | | | 0.43% | 1.06 (0.85-1.30) |
| ≥ 7 years | 23,965 | | 119 | | 0.50% | | 16,598 | | 93 | | | 0.56% | 1.19 (0.91-1.56) |
| Study cohort 2 | ACEI | | | | | | dCCB | | | | | | dCCB vs ACEI |
|  | N=373,601 | | | | | | N=373,601 | | | | | |  |
| Subgroups † | N | | Event, N | | (%) | | N | | Event, N | | | (%) | Adjusted HR (95% CI) ‡ |
| cDDD |  | |  | |  | |  | |  | | |  |  |
| < 500 cDDD | 154,094 | | 278 | | 0.18% | | 157,157 | | 294 | | | 0.19% | 1.26 (1.10-1.51) |
| 500-1500 cDDD | 128,768 | | 291 | | 0.23% | | 140,477 | | 411 | | | 0.29% | 1.32 (1.14-1.54) |
| 1500-3000 cDDD | 55,150 | | 156 | | 0.28% | | 54,889 | | 239 | | | 0.44% | 1.32 (1.08-1.60) |
| ≥ 3000 cDDD | 35,589 | | 169 | | 0.47% | | 21,078 | | 147 | | | 0.70% | 1.43 (1.22-1.63) |
| Duration |  | |  | |  | |  | |  | | |  |  |
| < 1 year | 137,949 | | 241 | | 0.17% | | 133,385 | | 253 | | | 0.19% | 1.29 (1.08-1.54) |
| 1-3 years | 149,399 | | 323 | | 0.22% | | 151,112 | | 402 | | | 0.27% | 1.32 (1.14-1.53) |
| 3-5 years | 53,567 | | 171 | | 0.32% | | 55,731 | | 223 | | | 0.40% | 1.28 (1.05-1.56) |
| 5-7 years | 20,895 | | 89 | | 0.43% | | 21,540 | | 123 | | | 0.57% | 1.32 (1.02-1.73) |
| ≥ 7 years | 11,791 | | 70 | | 0.59% | | 11,833 | | 90 | | | 0.76% | 1.34 (1.01-1.69) |
| Study cohort 3 | | ARB | | | | | | dCCB | | | | | dCCB vs ARB |
|  | | N=408,491 | | | | | | N=408,491 | | | | |  |
| Subgroups † | | N | | Event, N | | (%) | | N | | Event, N | (%) | | Adjusted HR (95% CI) ‡ |
| cDDD | |  | |  | |  | |  | |  |  | |  |
| < 500 cDDD | | 182,988 | | 275 | | 0.15% | | 174,689 | | 305 | 0.17% | | 1.18 (1.00-1.39) |
| 500-1500 cDDD | | 145,910 | | 358 | | 0.25% | | 154,107 | | 446 | 0.29% | | 1.21 (1.05-1.38) |
| 1500-3000 cDDD | | 57,309 | | 202 | | 0.35% | | 58,010 | | 274 | 0.47% | | 1.24 (1.04-1.49) |
| ≥ 3000 cDDD | | 22,284 | | 129 | | 0.58% | | 21,685 | | 141 | 0.65% | | 1.32 (1.07-1.54) |
| Duration | |  | |  | |  | |  | |  |  | |  |
| < 1 year | | 140,462 | | 189 | | 0.13% | | 149,607 | | 274 | 0.18% | | 1.22 (1.01-1.47) |
| 1-3 years | | 174,390 | | 417 | | 0.24% | | 166,561 | | 432 | 0.26% | | 1.10 (0.96-1.24) |
| 3-5 years | | 65,220 | | 224 | | 0.34% | | 58,384 | | 244 | 0.42% | | 1.25 (1.03-1.48) |
| 5-7 years | | 20,065 | | 82 | | 0.41% | | 22,014 | | 130 | 0.59% | | 1.38 (1.04-1.82) |
| ≥ 7 years | | 8,354 | | 53 | | 0.63% | | 11,925 | | 86 | 0.72% | | 1.08 (0.86-1.50) |
| Abbreviations. ACEI, angiotensin-converting enzyme inhibitor; ARB, angiotensin receptor blocker; cDDD, cumulative defined daily dose; CI, confidence interval; dCCB, dihydropyridine calcium channel blocker; HR, hazard ratio  † Due to the small number (<11) of kidney cancer incidence in each subgroup, we present the results with re-grouping the duration of use (≥ 7 years).  ‡ The HR was adjusted for Charlson comorbidity index. | | | | | | | | | | | | | |

Table S8. Baseline characteristics of non-dCCB versus ACEI and ARB

|  | Study cohort 4 | | | | Study cohort 5 | | | |
| --- | --- | --- | --- | --- | --- | --- | --- | --- |
|  | ACEI | | Non-dCCB | | ARB | | Non-dCCB | |
|  | N=65,110 | | N=65,110 | | N=70,211 | | N=70,211 | |
|  | N | % | N | % | N | % | N | % |
| Age (median, IQR) | 61 (54-72) | | 61 (53-70) | | 61 (53-71) | | 61 (53-70) | |
| < 50 years | 10,368 | 15.9% | 10,810 | 16.6% | 11,718 | 16.7% | 11,967 | 17.0% |
| 50-60 years | 17,476 | 26.8% | 18,183 | 27.9% | 19,120 | 27.2% | 19,620 | 27.9% |
| 60-70 years | 18,684 | 28.7% | 19,154 | 29.4% | 20,432 | 29.1% | 20,413 | 29.1% |
| ≥ 70 years | 18,582 | 28.5% | 16,963 | 26.1% | 18,941 | 27.0% | 18,211 | 25.9% |
| Sex |  |  |  |  |  |  |  |  |
| Men | 28,499 | 43.8% | 28,025 | 43.0% | 30,982 | 44.1% | 31,350 | 44.7% |
| Women | 36,611 | 56.2% | 37,085 | 57.0% | 39,229 | 55.9% | 38,861 | 55.3% |
| Geographical region |  |  |  |  |  |  |  |  |
| North | 10,412 | 16.0% | 10,725 | 16.5% | 12,750 | 18.2% | 11,169 | 15.9% |
| Northeast | 18,153 | 27.9% | 17,562 | 27.0% | 16,498 | 23.5% | 19,793 | 28.2% |
| Midwest | 26,547 | 40.8% | 27,233 | 41.8% | 29,012 | 41.3% | 28,915 | 41.2% |
| South | 9,148 | 14.1% | 8,536 | 13.1% | 11,151 | 15.9% | 9,199 | 13.1% |
| West | 850 | 1.3% | 1,054 | 1.6% | 800 | 1.1% | 1,135 | 1.6% |
| Insurance type |  |  |  |  |  |  |  |  |
| HMO | 7,745 | 11.9% | 7,167 | 11.0% | 8,503 | 12.1% | 7,886 | 11.2% |
| PPO | 34,019 | 52.2% | 35,792 | 55.0% | 36,660 | 52.2% | 38,770 | 55.2% |
| Others | 23,346 | 35.9% | 22,151 | 34.0% | 25,048 | 35.7% | 23,555 | 33.5% |
| Previous use of other AHTN | |  |  |  |  |  |  |  |
| BB | 15,894 | 24.4% | 13,726 | 21.1% | 16,258 | 23.2% | 14,969 | 21.3% |
| DU | 13,881 | 21.3% | 12,530 | 19.2% | 15,406 | 21.9% | 14,004 | 19.9% |
| Thiazide DU | 6,493 | 10.0% | 5,998 | 9.2% | 7,791 | 11.1% | 6,653 | 9.5% |
| Loop DU | 5,705 | 8.8% | 4,954 | 7.6% | 5,827 | 8.3% | 5,560 | 7.9% |
| K+ sparing DU | 3,162 | 4.9% | 2,862 | 4.4% | 3,378 | 4.8% | 3,191 | 4.5% |
| Comorbidities |  |  |  |  |  |  |  |  |
| Dyslipidemia | 40,679 | 62.5% | 39,771 | 61.1% | 43,464 | 61.9% | 42,607 | 60.7% |
| DM w/ complications | 6,175 | 9.5% | 5,789 | 8.9% | 6,442 | 9.2% | 6,243 | 8.9% |
| DM w/o complications | 17,248 | 26.5% | 16,627 | 25.5% | 18,100 | 25.8% | 18,012 | 25.7% |
| PVD | 7,962 | 12.2% | 7,050 | 10.8% | 8,367 | 11.9% | 7,705 | 11.0% |
| Ischemic stroke/TIA | 7,776 | 11.9% | 6,774 | 10.4% | 7,806 | 11.1% | 7,272 | 10.4% |
| Hemorrhagic stroke | 604 | 0.9% | 535 | 0.8% | 599 | 0.9% | 605 | 0.9% |
| Myocardial infarction | 3,439 | 5.3% | 3,033 | 4.7% | 3,638 | 5.2% | 3,459 | 4.9% |
| Angina | 1,397 | 2.1% | 1,302 | 2.0% | 1,490 | 2.1% | 1,361 | 1.9% |
| Heart failure | 8,205 | 12.6% | 7,404 | 11.4% | 8,499 | 12.1% | 8,322 | 11.9% |
| Atrial fibrillation | 19,740 | 30.3% | 19,459 | 29.9% | 20,258 | 28.9% | 21,019 | 29.9% |
| Chronic kidney disease | 972 | 1.5% | 1,088 | 1.7% | 1,109 | 1.6% | 1,067 | 1.5% |
| Renal failure | 160 | 0.2% | 169 | 0.3% | 166 | 0.2% | 186 | 0.3% |
| COPD | 5,274 | 8.1% | 4,772 | 7.3% | 5,599 | 8.0% | 5,261 | 7.5% |
| Other medication use |  |  |  |  |  |  |  |  |
| Lipid-lowering agents | 1,171 | 1.8% | 1,020 | 1.6% | 1,195 | 1.7% | 1,108 | 1.6% |
| Anti-diabetic agents | 9,577 | 14.7% | 9,291 | 14.3% | 10,141 | 14.4% | 10,090 | 14.4% |
| Anti-thrombotic agents | 12,017 | 18.5% | 11,467 | 17.6% | 13,543 | 19.3% | 13,217 | 18.8% |
| Anti-platelet agents | 3,937 | 6.0% | 3,969 | 6.1% | 4,591 | 6.5% | 4,652 | 6.6% |
| Anti-analgesic agents | 7,672 | 11.8% | 8,032 | 12.3% | 7,856 | 11.2% | 8,594 | 12.2% |
| Abbreviations: ACEI, angiotensin-converting enzyme inhibitor; ARB, angiotensin receptor blocker; AHTN, antihypertensive drugs; BB, beta-blocker; COPD, chronic obstructive pulmonary disease; Non-dCCB, non-dihydropyridine calcium channel blocker; DM, diabetes mellitus; DU, diuretic; MO, Health Maintenance Organization; IQR, interquartile range; PPO, preferred provider organization; PVD, peripheral vascular disease; TIA, transient ischemic attack | | | | | | | | |

Table S9. Risks of kidney cancer associated with non-dCCB versus ACEI and ARB

| Study cohort 4 | ACEI  (N=65,110) | | | | Non-dCCB | | | | Non-dCCB vs ACEI | |
| --- | --- | --- | --- | --- | --- | --- | --- | --- | --- | --- |
|  |  |  |  |  | (N=65,110) | | | |  |  |
|  | Event, N | (%) | PY | IR per 100,000 PY | Event, N | (%) | PY | IR per 100,000 PY | Unadjusted HR (95% CI) | Adjusted HR (95% CI) † |
|  | 169 | 0.26% | 144,300.0 | 117.1 | 196 | 0.30% | 166,397.1 | 117.8 | 1.16 (0.94-1.43) | 1.18 (0.95-1.45) |
| Study cohort 5 | ARB | | | | Non-dCCB | | | | Non-dCCB vs ARB | |
|  | (N=70,211) | | | | (N=70,211) | | | |  |  |
|  | Event, N | (%) | PY | IR per 100,000 PY | Event, N | (%) | PY | IR per 100,000 PY | Unadjusted HR (95% CI) | Adjusted HR (95% CI) † |
|  | 168 | 0.24% | 158,166.5 | 106.2 | 198 | 0.28% | 175,451.8 | 112.9 | 1.04 (0.85-1.28) | 1.06 (0.87-1.31) |
| Abbreviations. ACEI, angiotensin-converting enzyme inhibitor; ARB, angiotensin receptor blocker; Non-dCCB, non-dihydropyridine calcium channel blocker; PY, person-years; IR, incidence rate  † The model was adjusted for Charlson comorbidity index. | | | | | | | | | | |

Table S10. Results of subgroup analyses for kidney cancer risk compared non-dCCB to ACEI

|  | ACEI | | | Non-dCCB | | | Non-dCCB vs ACEI |  |
| --- | --- | --- | --- | --- | --- | --- | --- | --- |
|  | N=65,110 | | | N=65,110 | | |  |  |
| Subgroups † | N | Events, N | (%) | N | Events, N | (%) | Adjusted HR (95% CI) ‡ | p for difference |
| Age |  |  |  |  |  |  |  |  |
| < 50 years | 10,368 | 12 | 0.12% | 10,810 | 13 | 0.12% | 1.03 (0.47-2.24) |  |
| 50-60 years | 17,476 | 30 | 0.17% | 18,183 | 48 | 0.26% | 1.53 (0.97-2.40) | 0.706 |
| 60-70 years | 18,684 | 65 | 0.35% | 19,154 | 74 | 0.39% | 1.16 (0.83-1.62) |  |
| ≥ 70 years | 18,582 | 62 | 0.33% | 16,963 | 61 | 0.36% | 1.14 (0.80-1.61) |  |
| Sex |  |  |  |  |  |  |  |  |
| Male | 28,499 | 108 | 0.38% | 28,025 | 115 | 0.41% | 1.12 (0.86-1.46) |  |
| Female | 36,611 | 61 | 0.17% | 37,085 | 81 | 0.22% | 1.38 (0.98-1.90) | 0.346 |
| Myocardial infarction |  |  |  |  |  |  |  |  |
| No | 61,671 | 157 | 0.25% | 62,077 | 184 | 0.30% | 1.21 (0.98-1.5) |  |
| Yes | 3,439 | 12 | 0.35% | 3,033 | 12 | 0.40% | 1.23 (0.55-2.73) | 0.974 |
| Ischemic stroke/TIA |  |  |  |  |  |  |  |  |
| No | 57,334 | 147 | 0.26% | 58,336 | 170 | 0.29% | 1.18 (0.95-1.47) |  |
| Yes | 7,776 | 22 | 0.28% | 6,774 | 26 | 0.38% | 1.42 (0.81-2.51) | 0.551 |
| DM without complications |  |  |  |  |  |  |  |  |
| No | 47,862 | 107 | 0.22% | 48,483 | 121 | 0.25% | 1.16 (0.89-1.50) |  |
| Yes | 17,248 | 62 | 0.36% | 16,627 | 75 | 0.45% | 1.30 (0.93-1.83) | 0.582 |
| DM with complications |  |  |  |  |  |  |  |  |
| No | 58,935 | 148 | 0.25% | 59,321 | 163 | 0.27% | 1.14 (0.91-1.42) |  |
| Yes | 6,175 | 21 | 0.34% | 5,789 | 33 | 0.57% | 1.73 (1.00-2.99) | 0.166 |
| Heart failure |  |  |  |  |  |  |  |  |
| No | 56,905 | 142 | 0.25% | 57,706 | 171 | 0.30% | 1.24 (0.99-1.54) |  |
| Yes | 8,205 | 27 | 0.33% | 7,404 | 25 | 0.34% | 1.08 (0.62-1.84) | 0.642 |
| Atrial fibrillation |  |  |  |  |  |  |  |  |
| No | 45,370 | 105 | 0.23% | 45,651 | 141 | 0.31% | 1.36 (1.06-1.76) |  |
| Yes | 19,740 | 64 | 0.32% | 19,459 | 55 | 0.28% | 0.95 (0.66-1.36) | 0.106 |
| Dyslipidemia |  |  |  |  |  |  |  |  |
| No | 24,431 | 54 | 0.22% | 25,339 | 66 | 0.26% | 1.18 (0.82-1.69) |  |
| Yes | 40,679 | 115 | 0.28% | 39,771 | 130 | 0.33% | 1.22 (0.94-1.56) | 0.858 |
| Peripheral vascular disease |  |  |  |  |  |  |  |  |
| No | 57,148 | 135 | 0.24% | 58,060 | 160 | 0.28% | 1.21 (0.97-1.50) |  |
| Yes | 7,962 | 34 | 0.43% | 7,050 | 36 | 0.51% | 1.24 (0.78-2.00) | 0.886 |
| Chronic kidney disease |  |  |  |  |  |  |  |  |
| No | 59,836 | 145 | 0.24% | 60,338 | 165 | 0.27% | 1.17 (0.94-1.46) |  |
| Yes | 5,274 | 24 | 0.46% | 4,772 | 31 | 0.65% | 1.46 (0.86-2.48) | 0.442 |
| COPD |  |  |  |  |  |  |  |  |
| No | 55,533 | 136 | 0.24% | 55,819 | 162 | 0.29% | 1.23 (0.98-1.54) |  |
| Yes | 9,577 | 33 | 0.34% | 9,291 | 34 | 0.37% | 1.14 (0.70-1.83) | 0.761 |
| Abbreviations: ACEI, angiotensin-converting enzyme inhibitor; ARB, angiotensin receptor blocker; CI, confidence interval; COPD, chronic obstructive pulmonary disease; HR, hazard ratio; non-dCCB, non-dihydropyridine calcium channel blocker; TIA, transient ischemic attack  † Due to the small number (<11) of kidney cancer incidence in subgroups of hemorrhagic stroke, angina, and renal failure, we were unable to present the data.  ‡ The model was adjusted for Charlson comorbidity index. | | | | | | | | |

Table S11. Results of subgroup analyses for kidney cancer risk compared non-dCCB to ARB

|  | ARB | | | dCCB | | | dCCB vs ARB |  |
| --- | --- | --- | --- | --- | --- | --- | --- | --- |
|  | N=373,601 | | | N=373,601 | | |  |  |
| Subgroups † | N | Events, N | (%) | N | Events, N | (%) | Adjusted HR (95% CI) ‡ | p for difference |
| Age |  |  |  |  |  |  |  |  |
| < 60 years | 30,838 | 49 | 0.16% | 31,587 | 54 | 0.17% | 1.12 (0.82-1.56) |  |
| 60-70 years | 20,432 | 54 | 0.26% | 20,413 | 76 | 0.37% | 1.26 (0.89-1.77) | 0.244 |
| ≥ 70 years | 18,941 | 65 | 0.34% | 18,211 | 68 | 0.37% | 1.01 (0.72-1.40) |  |
| Sex |  |  |  |  |  |  |  |  |
| Male | 30,982 | 98 | 0.32% | 31,350 | 118 | 0.38% | 1.08 (0.82-1.41) |  |
| Female | 39,229 | 70 | 0.18% | 38,861 | 80 | 0.21% | 1.06 (0.78-1.45) | 0.929 |
| Myocardial infarction |  |  |  |  |  |  |  |  |
| No | 66,573 | 155 | 0.23% | 66,752 | 179 | 0.27% | 1.03 (0.83-1.28) |  |
| Yes | 3,638 | 13 | 0.36% | 3,459 | 19 | 0.55% | 1.54 (0.76-3.13) | 0.286 |
| Ischemic stroke/TIA |  |  |  |  |  |  |  |  |
| No | 62,405 | 147 | 0.24% | 62,939 | 174 | 0.28% | 1.05 (0.84-1.31) |  |
| Yes | 7,806 | 21 | 0.27% | 7,272 | 24 | 0.33% | 1.18 (0.66-2.12) | 0.710 |
| DM without complications |  |  |  |  |  |  |  |  |
| No | 52,111 | 111 | 0.21% | 52,199 | 125 | 0.24% | 1.02 (0.79-1.29) |  |
| Yes | 18,100 | 57 | 0.31% | 18,012 | 73 | 0.41% | 1.19 (0.84-1.68) | 0.436 |
| DM with complications |  |  |  |  |  |  |  |  |
| No | 63,769 | 140 | 0.22% | 63,968 | 161 | 0.25% | 1.03 (0.82-1.29) |  |
| Yes | 6,442 | 28 | 0.43% | 6,243 | 37 | 0.59% | 1.30 (0.79-2.12) | 0.398 |
| Heart failure |  |  |  |  |  |  |  |  |
| No | 61,712 | 139 | 0.23% | 61,889 | 164 | 0.26% | 1.04 (0.82-1.30) |  |
| Yes | 8,499 | 29 | 0.34% | 8,322 | 34 | 0.41% | 1.18 (0.72-1.94) | 0.670 |
| Atrial fibrillation |  |  |  |  |  |  |  |  |
| No | 49,953 | 100 | 0.20% | 49,192 | 134 | 0.27% | 1.18 (0.91-1.53) |  |
| Yes | 20,258 | 68 | 0.34% | 21,019 | 64 | 0.30% | 0.90 (0.64-1.26) | 0.213 |
| Dyslipidemia |  |  |  |  |  |  |  |  |
| No | 26,747 | 53 | 0.20% | 27,604 | 63 | 0.23% | 1.01 (0.70-1.44) |  |
| Yes | 43,464 | 115 | 0.26% | 42,607 | 135 | 0.32% | 1.10 (0.86-1.40) | 0.680 |
| Peripheral vascular disease |  |  |  |  |  |  |  |  |
| No | 61,844 | 150 | 0.24% | 62,506 | 155 | 0.25% | 0.91 (0.73-1.14) |  |
| Yes | 8,367 | 18 | 0.22% | 7,705 | 43 | 0.56% | 1.49 (0.84-3.33) | 0.160 |
| Chronic kidney disease |  |  |  |  |  |  |  |  |
| No | 64,612 | 143 | 0.22% | 64,950 | 169 | 0.26% | 1.05 (0.84-1.31) |  |
| Yes | 5,599 | 25 | 0.45% | 5,261 | 29 | 0.55% | 1.17 (0.68-2.01) | 0.725 |
| COPD |  |  |  |  |  |  |  |  |
| No | 60,070 | 136 | 0.23% | 60,121 | 164 | 0.27% | 1.07 (0.85-1.34) |  |
| Yes | 10,141 | 32 | 0.32% | 10,090 | 34 | 0.34% | 1.03 (0.63-1.67) | 0.864 |
| Abbreviations: ACEI, angiotensin-converting enzyme inhibitor; ARB, angiotensin receptor blocker; CI, confidence interval; COPD, chronic obstructive pulmonary disease; HR, hazard ratio; non-dCCB, non-dihydropyridine calcium channel blocker; TIA, transient ischemic attack  † Due to the small number (<11) of kidney cancer incidence in subgroups of hemorrhagic stroke, angina, and renal failure, we were unable to present the data. Also, we re-grouped age categories (< 60 years).  ‡ The model was adjusted for Charlson comorbidity index. | | | | | | | | |

Table S12. Results of exposure to the other AHTN including ACEI, ARB, and non-dCCB with the risk of kidney cancer

| Study cohort 4 | ACEI | | | Non-dCCB | | | Non-dCCB vs ACEI |
| --- | --- | --- | --- | --- | --- | --- | --- |
|  | N=65,110 | | | N=65,110 | | |  |
| Subgroups † | N | Event, N | (%) | N | Event, N | (%) | Adjusted HR (95% CI) ‡ |
| cDDD |  |  |  |  |  |  |  |
| < 500 cDDD | 29,056 | 57 | 0.20% | 37,060 | 69 | 0.19% | 1.10 (0.77-1.56) |
| 500-1500 cDDD | 21,512 | 49 | 0.23% | 21,820 | 86 | 0.39% | 1.42 (0.91-2.01) |
| 1500-2500 cDDD | 6,918 | 27 | 0.39% | 4,641 | 24 | 0.52% | 0.94 (0.54-1.63) |
| ≥ 2500 cDDD | 7,624 | 36 | 0.47% | 1,589 | 17 | 1.07% | 1.64 (0.92-2.94) |
| Duration |  |  |  |  |  |  |  |
| < 1 year | 25,412 | 38 | 0.15% | 21,372 | 33 | 0.15% | 0.99 (0.72-1.36) |
| 1-3 years | 26,003 | 74 | 0.28% | 27,058 | 70 | 0.26% | 1.27 (0.80-2.03) |
| 3-5 years | 8,722 | 28 | 0.32% | 10,455 | 56 | 0.54% | 1.74 (0.85-2.76) |
| ≥ 5 years | 4,973 | 29 | 0.58% | 6,225 | 37 | 0.59% | 1.08 (0.66-1.75) |
| Study cohort 5 | ARB | | | Non-dCCB | | | Non-dCCB vs ARB |
|  | N=70,211 | | | N=70,211 | | |  |
| Subgroups † | N | Event, N | (%) | N | Event, N | (%) | Adjusted HR (95% CI) ‡ |
| cDDD |  |  |  |  |  |  |  |
| < 500 cDDD | 32,503 | 51 | 0.16% | 40,788 | 72 | 0.18% | 1.03 (0.72-1.47) |
| 500-1500 cDDD | 24,788 | 60 | 0.24% | 22,983 | 88 | 0.38% | 1.24 (0.89-1.73) |
| 1500-2500 cDDD | 7,369 | 30 | 0.41% | 4,761 | 22 | 0.46% | 0.81 (0.46-1.40) |
| ≥ 2500 cDDD | 5,551 | 27 | 0.49% | 1,679 | 16 | 0.95% | 1.32 (0.70-2.47) |
| Duration |  |  |  |  |  |  |  |
| < 1 year | 24,928 | 39 | 0.16% | 23,779 | 36 | 0.15% | 0.86 (0.55-1.34) |
| 1-3 years | 30,037 | 79 | 0.26% | 29,174 | 73 | 0.25% | 0.93 (0.68-1.28) |
| 3-5 years | 10,757 | 34 | 0.32% | 10,794 | 48 | 0.44% | 1.39 (0.90-2.16) |
| ≥ 5 years | 4,489 | 16 | 0.36% | 6,464 | 41 | 0.63% | 1.60 (0.89-2.84) |
| Abbreviations. ACEI, angiotensin-converting enzyme inhibitor; ARB, angiotensin receptor blocker; cDDD, cumulative defined daily dose; CI, confidence interval; HR, hazard ratio; hazard ratio; non-dCCB, non-dihydropyridine calcium channel blocker  † Due to the small number (<11) of kidney cancer incidence in each subgroups, we re-grouped the cDDD (1500-2500 cDDD and ≥ 2500 cDDD) and duration of use (≥ 5 years).  ‡ The model was adjusted for Charlson comorbidity index. | | | | | | | |

Table S13. Results of sensitivity analyses for kidney cancer risk compared non-dCCB to ACEI and ARB

| Study cohort 4 | ACEI | | | Non-dCCB | | | Non-dCCB vs ACEI |
| --- | --- | --- | --- | --- | --- | --- | --- |
|  | N | Event, N | (%) | N | Event, N | (%) | Adjusted HR (95% CI) † |
| Main analysis | 65,110 | 169 | 0.26% | 65,110 | 196 | 0.30% | 1.18 (0.95-1.45) |
| Redefinition of new AHTN use | 35,795 | 89 | 0.25% | 37,784 | 112 | 0.30% | 1.20 (0.92-1.58) |
| Long-term use (1000+ cDDD) | 15,755 | 63 | 0.40% | 19,141 | 102 | 0.53% | 1.36 (0.98-1.87) |
| Lag time (2 years) | 65,034 | 93 | 0.14% | 65,020 | 106 | 0.16% | 1.19 (0.90-1.58) |
| Without compelling indications | 27,725 | 49 | 0.18% | 27,907 | 65 | 0.23% | 1.30 (0.90-1.92) |
| As-treated approach | 65,110 | 110 | 0.17% | 65,110 | 149 | 0.23% | 1.05 (0.86-1.29) |
| Without propensity score matching | 749,990 | 1,616 | 0.22% | 67,887 | 202 | 0.30% | 1.20 (1.04-1.40) |
| Study cohort 5 | ARB | | | Non-dCCB | | | Non-dCCB vs ARB |
|  | N | Event, N | (%) | N | Event, N | (%) | Adjusted HR (95% CI) † |
| Main analysis | 70,211 | 168 | 0.24% | 70,211 | 198 | 0.28% | 1.06 (0.87-1.31) |
| Redefinition of new AHTN use | 35,139 | 76 | 0.22% | 36,684 | 100 | 0.27% | 1.13 (0.84-1.52) |
| Long-term use (1000+ cDDD) | 17,482 | 66 | 0.38% | 19,818 | 97 | 0.49% | 1.18 (0.88-1.62) |
| Lag time (2 years) | 70,126 | 83 | 0.12% | 70,112 | 99 | 0.14% | 0.97 (0.72-1.30) |
| Without compelling indications | 31,440 | 52 | 0.17% | 30,077 | 59 | 0.20% | 1.07 (0.83-1.48) |
| As-treated approach | 70,211 | 109 | 0.16% | 70,211 | 155 | 0.22% | 1.09 (0.88-1.31) |
| Without propensity score matching | 727,065 | 1,783 | 0.25% | 73,160 | 207 | 0.28% | 0.97 (0.83-1.12) |
| Abbreviations. ACEI, angiotensin-converting enzyme inhibitor; ARB, angiotensin receptor blocker; cDDD, cumulative defined daily dose; CI, confidence interval; HR, hazard ratio; non-dCCB, non-dihydropyridine calcium channel blocker  † For sensitivity analysis, the model was adjusted for all confounders, which were used in the matching model in the main study, to account for potential imbalance between two groups. | | | | | | | |

Figure S1. Flowchart for the selection of study participants of new users of ACEI, ARB, and CCB

Figure S1 Legend.

To reduce potential indication bias, we included patients with a diagnosis record of hypertension who received the first treatment options of AHTN including ACEI, ARB, and dCCB. After applying inclusion and exclusion criteria, we assembled five propensity-score matched (1:1 ratio) cohorts: ACEI+ARB; ACEI+dCCB; ARB+dCCB; ACEI+non-dCCB; and ARB+non-dCCB.

Abbreviations. ACEI, angiotensin-converting enzyme inhibitor; AHTN, antihypertensive drugs; ARB, angiotensin receptor blocker; dCCB, dihydropyridine calcium channel blocker; PKD, polycystic kidney disease; PSM, propensity score matching; TS, tuberous sclerosis; VHL, Von Hippel-Lindau
